# Supplementary material for: Human variation impacting MCOLN2 restricts Salmonella Typhi replication by magnesium deprivation
Source: Cell Genom. 2023 Apr 4;3(5):100290. doi: 10.1016/j.xgen.2023.100290 (PMC10203047; doi:10.1016/j.xgen.2023.100290)
Supplement: Document S1. Figures S1–S4 and Tables S3 and S4 [file mmc1.pdf]

Cell Genomics, Volume *3*

Supplemental information

Human variation impacting *MCOLN2* restricts *Salmonella* Typhi replication by magnesium deprivation

Kyle D. Gibbs, Liuyang Wang, Zhuo Yang, Caroline E. Anderson, Jeffrey S. Bourgeois, Yanlu Cao, Margaret R. Gaggioli, Martin Biel, Rosa Puertollano, Cheng-Chang Chen, and Dennis C. Ko

A

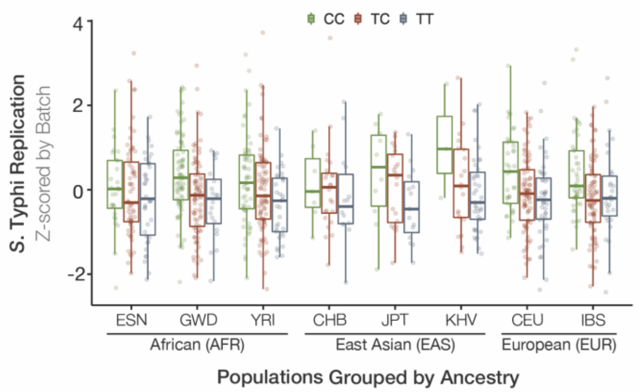

Populations in Hi-HoST Cellular GWAS

Ancestry	Pop	Description	N	N
AFR	ESN	Esan in Nigeria	129	486
	GWD	Gambian Mandinka in Western Division, The Gambia	179	
	YRI	Yoruba in Ibadan, Nigeria	178	
EAS	CHB	Han Chinese in Beijing, China	45	152
	JPT	Japanese in Tokyo, Japan	45	
	KHV	Kihn Vietnamese in Ho Chi Minh City, Vietnam	62	
EUR	CEU	Utah residents with N & W European ancestry	166	323
	IBS	Iberians in Spain	157	

B

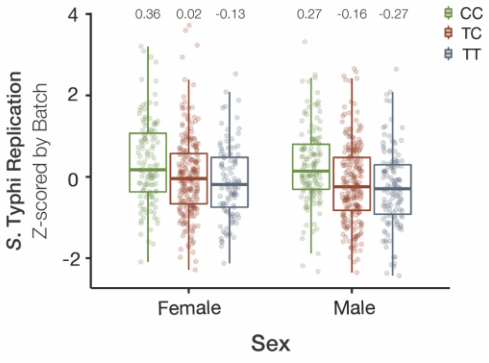

Replciation Regressions Stratified by Sex

	Female	Male	All
N	460	501	961
Slope	-0.25	-0.28	-0.26
SE of Slope	±0.06	±0.06	±0.04
p value	1.3×10^{-4}	3.2×10^{-6}	1.7×10^{9}
R^2	0.029	0.041	0.036

Figure S1. The rs10873679 C-allele associates with more *S.* Typhi replication across all sampled populations and in both sexes, related to Figure 1. (**A**) Increased z-scored *S.* Typhi replication associated with the C-allele in all populations. Individual population regressions slopes range from -0.12 in ESN to -0.40 in GWD. Table below lists number of LCLs from each population used in cellular GWAS. (**B**) The rs10873679 C-allele associated with more z-scored *S.* Typhi replication in both sexes. Linear regressions are listed in the table. A two-way ANOVA with genotype and sex finds no significant interaction between sex & *MCOLN2* genotype ($p = 0.8$) despite both individual factors being a significant source of variation (genotype $p=1.9 \times 10^{-9}$ & sex $p=0.02$).

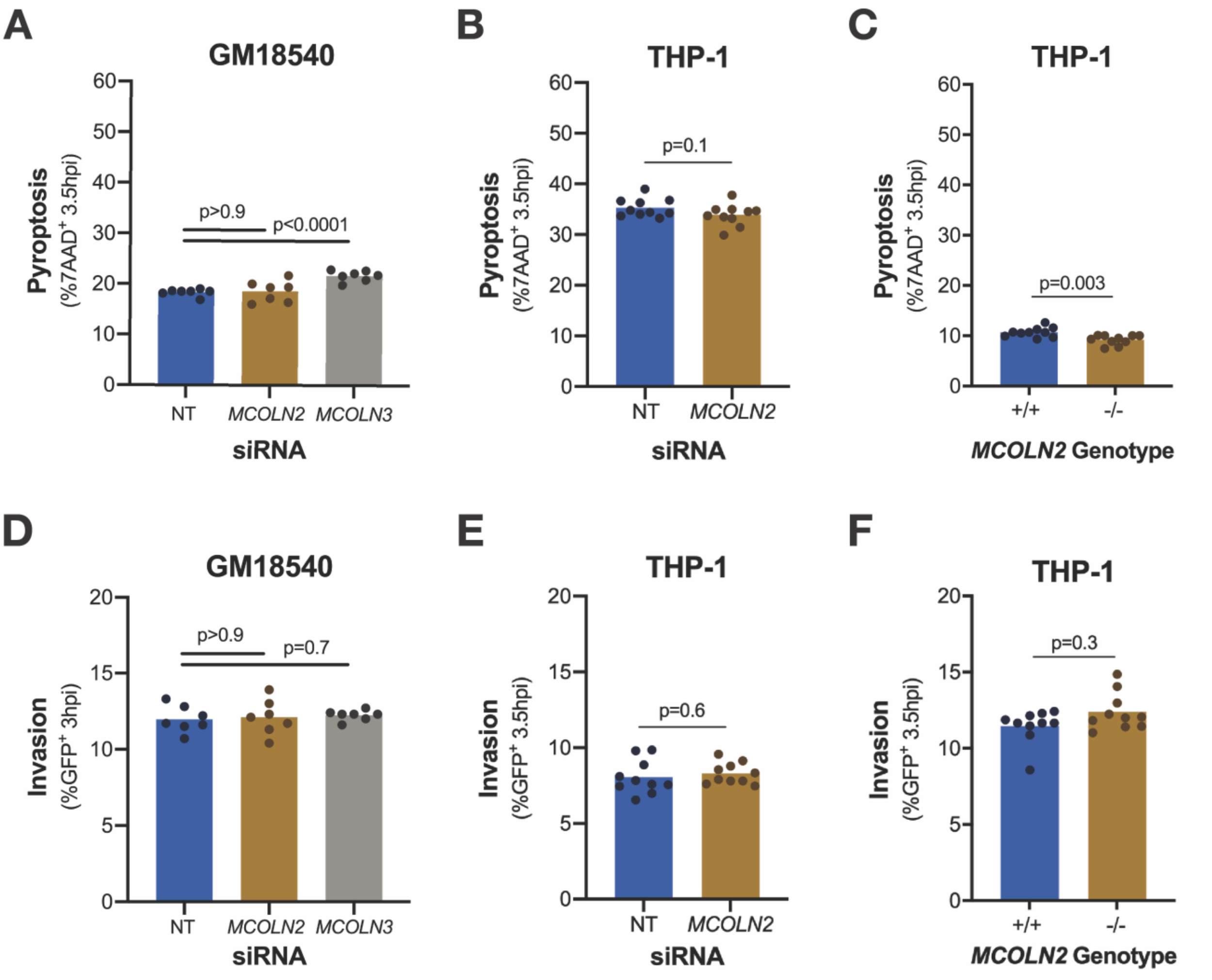

Figure S2. Neither *MCOLN2* knockdown nor knockout affect pyroptosis or invasion during *S.* Typhi infection in Figure 2E-G. (**A–C**) Pyroptosis is the percent of cells dead (7AAD$^+$) at 3.5 hpi. (**D–E**) Invasion is the percent of living (7AAD$^-$) and infected (GFP$^+$) cells at 3.5 hpi. LCL (GM18540) infection in A & D are nine replicates from three experiments from Figure 2E. THP-1 infection in B, C, E, & F are ten replicates from two experiments presented in Figure 2F & G. In A & F, the p values are from Mann-Whitney U tests. In B-E, p values are from unpaired *t* tests. While wild-type and knockout cells significantly differ in C, the effect size is only 0.5% respectively.

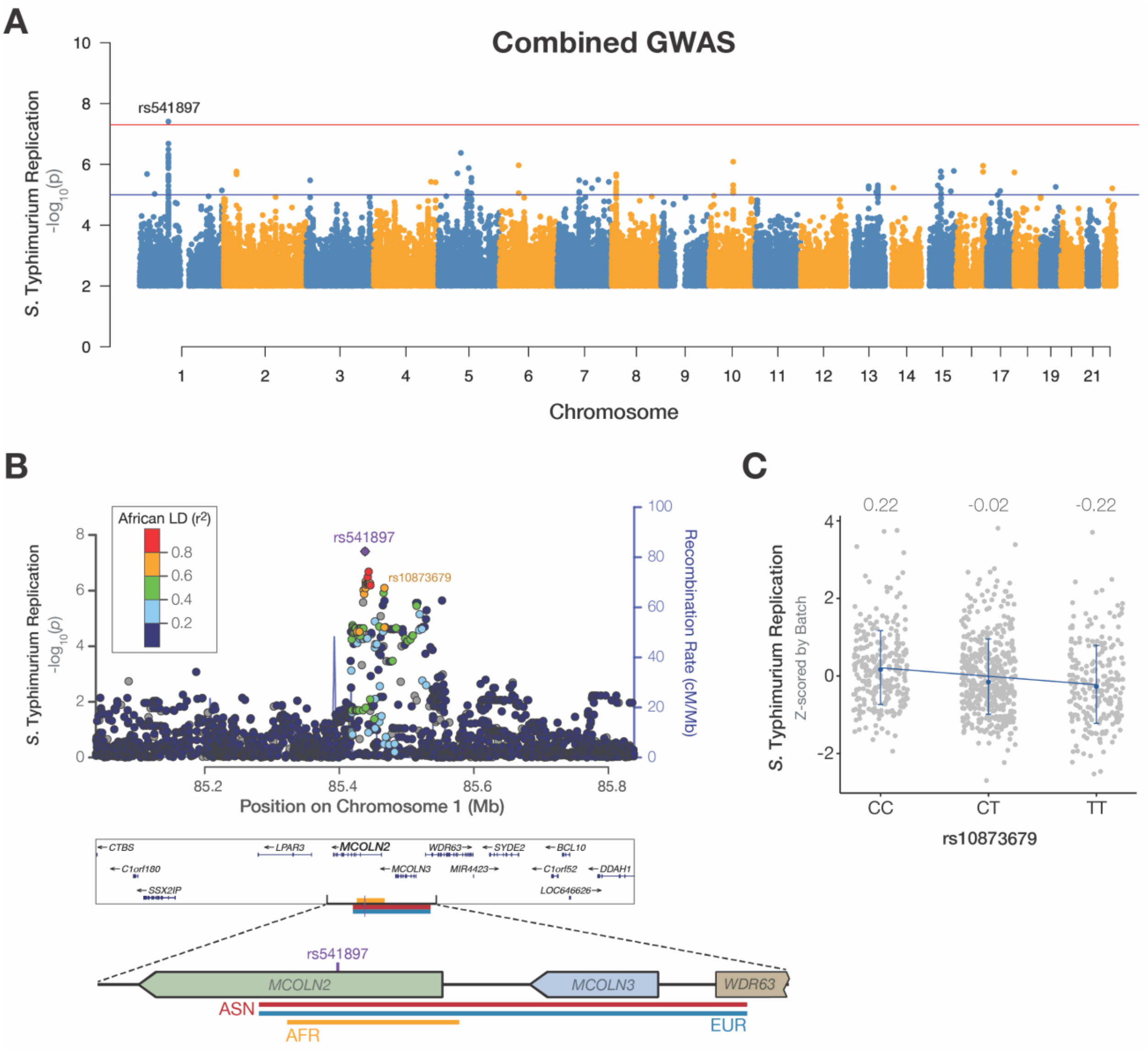

Figure S3. Increased *S.* Typhimurium replication also associates with the rs10873679 C- allele, related to Figure 2H. **(A)** The rs541897 locus association with z-scored *S.* Typhimurium replication (p = 3.9×10^{-8}) passes genome-wide significance threshold (5×10^{-8}; red line). **(B)** A local Manhattan plot of the *S.* Typhimurium replication-associated locus on chromosome 1 (hg19 build) shows that the *S.* Typhimurium lead SNP, rs541897, is in the same locus as the *S.* Typhi lead SNP, rs10873679. The *S.* Typhi and *S.* Typhimurium lead SNPs are linked (LD $r^2 = 0.71$ in African ancestry populations). Dots for each SNP are color coded by African (AFR) linkage disequilibrium (LD; r^2) from 1000 genomes Nov 2014 release. A 164 kb zoom in on the *MCOLN2/3* region indicates regions well-linked (r^2>0.6) with rs541897 by continental ancestry: African with a 41 kb orange bar, European (EUR) with a 116 kb blue bar, & Asian (ASN) with a 116 kb red bar. **(C)** The rs10873679 C-allele associates with more *S.* Typhimurium replication. The slope of a linear regression predicting replication from genotype is significantly different from zero ($\beta = -0.22 \pm 0.04$; $p = 3.5 \times 10^{-6}$). Mean replication of each genotype is listed above their respective dot plot. Bars are mean ±SD.

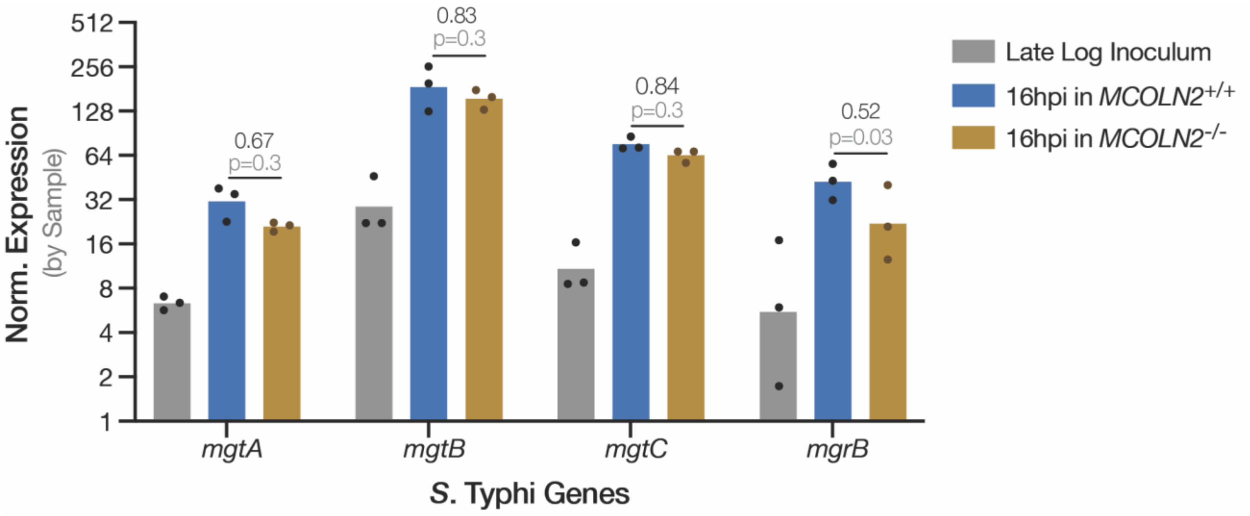

Figure S4. The increased expression of Mg^{2+}-regulated *S.* Typhi genes inside THP-1 monocytes is consistently decreased in *MCOLN2* knockouts, same data underlying log_2[Fold Change] in Figure 3D. Three replicates are from three experiments. Bars are geometric mean. Inoculum RNA was mixed with RNA from uninfected THP-1 cells of each genotype, so each late log replicate is the average of two technical (sequencing) replicates. Ratios (black text) are geometric mean from expression of bacteria replicating inside *MCOLN2* knockout over bacteria replicating in wild-type THP-1 cells. P values (grey) are from paired t tests corrected for multiple testing by Holm-Šídák method.

Table S3. *Salmonella enterica* ser. Typhi strain Ty2 gene sets used in GSEA. Related to Figures 3C and 3D.

Gene Sets	N	Genes																							
**Housekeeping**	**9**	*rpoD*	*aroC*	*dnaN*	*hemD*	*hisD*	*sucA*	*thrA*	*dnaK*	*rpoA*															
**SPI-1**	**24**	*sicP*	*sptP*	*iacP*	*prgH*	*invA*	*sigE*	*invF*	*spaM*	*prgI*	*invF*	*sipA*	*sipB*	*sipC*	*sipD*	*spaS*	*spaR*	*spaP*	*spaO*	*hilA*	*hilD*	*spaK*	*spaJ*	*spaI*	*spaH*
**SPI-2**	**23**	*spiC*	*spiA*	*ssaD*	*ssaE*	*sseA*	*sseB*	*sscA*	*sseC*	*sseD*	*sseE*	*sscB*	*sseF*	*sseG*	*ssaG*	*ssaI*	*ssaJ*	*ssaK*	*ssaL*	*ssaR*	*ssaR*	*ssaS*	*ssaT*	*ssaU*	
**PhoP Regulon**	**19**	*pmrD*	*mgtA*	*phoN*	*phoP*	*phoQ*	*mgtB*	*virK*	*ybjX*	*mgrB*	*pagP*	*pagD*	*iraP*	*pagC*	*mgtC*	*pipD*	*pagK*	*ugtL*	*pgtE*	*spiC*					
**Magnesium Influx**	**4**	*mgtA*	*mgtB*	*mgtS*	*mgrB*																				
**PmrA Regulon**	**11**	*pmrC*	*pmrA*	*pmrB*	*naxD*	*arnB*	*arnC*	*arnA*	*arnD*	*arnT*	*arnE*	*arnF*													
**Zinc Efflux**	**4**	*fieF*	*zntA*	*zntB*	*zitB*																				
**Zinc Influx**	**4**	*zupT*	*znuA*	*znuB*	*znuC*																				
**Ferrous Iron Efflux**	**2**	*fieF*	*iceT*																						
**Ferrous Iron Influx**	**3**	*feoA*	*feoB*	*feoC*																					
**Manganese Influx**	**7**	*mntH*	*sitA*	*sitB*	*sitC*	*sitD*	*mntR*	*mntS*																	
**Copper Response**	**8**	*copA*	*cueR*	*cueO*	*cueP*	*scsA*	*scsB*	*scsC*	*scsD*																
**MFS Efflux**	**5**	*mdtH*	*mdtM*	*mdtG*	*emrD*	*mdfA*																			
**TolC Efflux**	**5**	*macB*	*tolC*	*acrA*	*acrE*	*marA*																			
**RpoE Regulon**	**5**	*rpoE*	*rseA*	*rseB*	*rseC*																				

Table S4. Oligonucleotides grouped by function in study. Related to STAR Methods.

Lambda-Red Recombination

Target Gene	Gene ID	Primer Designations	Fwd for Kan^R Cassette Generation	Rvr for Kan^R Cassette Generation
mgtA	t4491	DK972 & DK973	TATAATCCGCGGCGCAAATTATTTACTTACCGGAGGCGAC gtgtaggctggagctgcttc	TCGGGGATTAAGCACGCTGGCGAATCCCCGACGAAAGTGT catatgaatatcctccttag
mgtB	t3755	DK974 & DK975	ATATGCAGGAAACACTACACCTTAATTTTGGGGATTCATC gtgtaggctggagctgcttc	TATCGGGTGAGCGATTCATCTGGGCGATCCTCAAACATTA catatgaatatcctccttag
ssaT	t1289	DK3 & DK433	tacccggcagataatgttacgaattggagagcatggttga gtgtaggctggagctgcttc	TCACGTAATTTCTTTTCTGTAGGCTGTTCTGTTTTCTCGC catatgaatatcctccttag

Deletion Confirmation

Target Gene(s)	Gene ID	Primer Designations	Fwd	Rvr
phoPQ	t1689 & t1690	DK1008 & DK1009	CATGACGCCGGCAAATTATATC	GAAAGTCGGGCCAGTTAAGA
ssaT	t1289	DK434 & DK435	CGGTAGTTGGTGTCATCGTAAG	AGCGCAATCAGCTGAAATAATG
mgtA	t4491	DK980 & DK981	CGTGACGCTGATGGTGATAAA	ACATCTCCTCCTCTCGTTCTG
mgtB	t3755	DK982 & DK983	CAGGCGTATAAGGAGGGAATG	ACACCAACAGGCTAATCAGTAA
MCOLN2	ENSG00000153898	DK859 & DK860	CTGGGGTAATTTTCCAAAAGCAGT	AGGTTATTCTTCTGTGGGACTTGT

SYBR RT-qPCR

Target Gene(s)	Gene ID	Primer Designations	Fwd	Rvr
ACTB	ENSG00000075624	DK880 & DK881	CCTGTACGCCAACACAGTGC	ATACTCCTGCTTGCTGATCC
rpoD	t3131	DK628 & DK629	GAAATGGGCACTGTTGAACTG	CAGATAGGTAATGGCTTCCGG
