# Supplementary material for: Human variation impacting MCOLN2 restricts Salmonella Typhi replication by magnesium deprivation
Source: Cell Genom. 2023 Apr 4;3(5):100290. doi: 10.1016/j.xgen.2023.100290 (PMC10203047; doi:10.1016/j.xgen.2023.100290)
Supplement: Document S2. Article plus supplemental information [file mmc4.pdf]

Cell Genomics

Article

Human variation impacting *MCOLN2* restricts *Salmonella* Typhi replication by magnesium deprivation

Graphical abstract

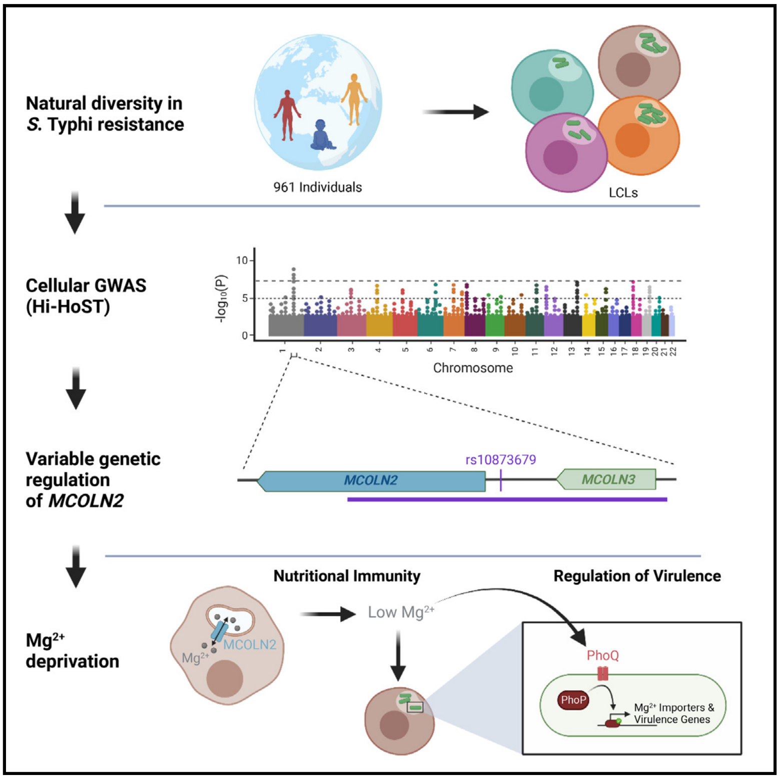

Highlights

- Cellular GWAS revealed *MCOLN2* as an inhibitor of *S.* Typhi intracellular replication
- MCOLN2 acts as an inward rectifying Mg^{2+} channel on endolysosomes
- MCOLN2 deprives *S.* Typhi of Mg^{2+} to restrict growth
- *S.* Typhi replication in *MCOLN2* knockout cells depends on bacterial PhoPQ signaling

Authors

Kyle D. Gibbs, Liuyang Wang, Zhuo Yang, ..., Rosa Puertollano, Cheng-Chang Chen, Dennis C. Ko

Correspondence

dennis.ko@duke.edu

In brief

Gibbs et al. conducted a cellular GWAS of nearly a thousand lymphoblastoid cells to identify genetic variation affecting the MCOLN2 (TRPML2) divalent cation channel as the primary genetic determinant of variation in *Salmonella* Typhi intracellular replication. MCOLN2 deprives *S.* Typhi of Mg^{2+}, serving as an important mechanism of nutritional immunity.

Gibbs et al., 2023, Cell Genomics *3*, 100290
May 10, 2023 © 2023 The Author(s).
https://doi.org/10.1016/j.xgen.2023.100290

CellPress

Cell Genomics

CellPress
OPEN ACCESS

Article

Human variation impacting *MCOLN2* restricts *Salmonella* Typhi replication by magnesium deprivation

Kyle D. Gibbs,[1] Liuyang Wang,[1] Zhuo Yang,[2] Caroline E. Anderson,[1] Jeffrey S. Bourgeois,[1,3] Yanlu Cao,[1] Margaret R. Gaggioli,[1] Martin Biel,[2] Rosa Puertollano,[4] Cheng-Chang Chen,[5,6] and Dennis C. Ko[1,3,7,8,*]

[1]Department of Molecular Genetics and Microbiology, School of Medicine, Duke University, Durham, NC 27710, USA
[2]Department of Pharmacy, Center for Drug Research, Ludwig-Maximilians-Universität München, Munich, Germany
[3]University Program in Genetics and Genomics, Duke University, Durham, NC 27710, USA
[4]Cell and Developmental Biology Center, National Heart, Lung, & Blood Institute, NIH, Bethesda, MD 20892, USA
[5]Department of Clinical Laboratory Sciences and Medical Biotechnology, College of Medicine, National Taiwan University, Taipei 100, Taiwan
[6]Department of Laboratory Medicine, National Taiwan University Hospital, Taipei 100, Taiwan
[7]Division of Infectious Diseases, Department of Medicine, School of Medicine, Duke University, Durham, NC 27710, USA
[8]Lead contact
*Correspondence: dennis.ko@duke.edu
https://doi.org/10.1016/j.xgen.2023.100290

SUMMARY

Human genetic diversity can reveal critical factors in host-pathogen interactions. This is especially useful for human-restricted pathogens like *Salmonella enterica* serovar Typhi (*S.* Typhi), the cause of typhoid fever. One key defense during bacterial infection is nutritional immunity: host cells attempt to restrict bacterial replication by denying bacteria access to key nutrients or supplying toxic metabolites. Here, a cellular genome-wide association study of intracellular replication by *S.* Typhi in nearly a thousand cell lines from around the world—and extensive follow-up using intracellular *S.* Typhi transcriptomics and manipulation of magnesium availability—demonstrates that the divalent cation channel mucolipin-2 (MCOLN2 or TRPML2) restricts *S.* Typhi intracellular replication through magnesium deprivation. Mg^{2+} currents, conducted through MCOLN2 and out of endolysosomes, were measured directly using patch-clamping of the endolysosomal membrane. Our results reveal Mg^{2+} limitation as a key component of nutritional immunity against *S.* Typhi and as a source of variable host resistance.

INTRODUCTION

Genome-wide association studies (GWASs) are a powerful method to identify common genetic variants associated with risk, resistance, or other quantitative measures of infectious disease.[1] However, connecting variants identified by whole-organism GWAS to disease pathogenesis is often challenging—especially when it is unclear how the identified variants affect nearby genes or how these genes relate to the disease under investigation. To solve this, studies look among the disease-linked variants for those that associate with expression of nearby genes (called expression quantitative trait loci [eQTLs]), which can provide important clues, especially during stimulation with pathogens[2] or pathogen-associated molecular patterns.[3,4] A complimentary approach to connect variant and disease is GWAS of cellular traits, such as our Hi-HOST (high-throughput human *in vitro* susceptibility testing)[5,6] platform, which associates genetic variation with quantifiable cellular traits, such as invasion,[7] inflammation,[8] and intracellular pathogen replication. As a further benefit, cellular GWASs provide control of environmental and pathogen variation, which boosts statistical power by reducing noise. Used together, cellular GWASs of eQTLs can connect genetic variants to both altered gene expression and cellular process, explaining how the identified variation impacts clinical outcomes and facilitating subsequent mechanistic studies.

Here, we used this approach to study susceptibility to the human-restricted enteric pathogen *Salmonella enterica* ser. Typhi (*S.* Typhi), which relies on a permissive niche inside immune cells to cause the life-threatening syndrome of typhoid fever.[9] We discovered that the interferon-inducible[10] host cation channel, mucolipin-2 (MCOLN2 or TRPML2), is critical for nutritional immunity against *S.* Typhi. In the dynamic competition between the host and bacteria, nutritional immunity is the ongoing effort of the host cell to restrict *Salmonella* replication by depriving it of key nutrients[11–13] or delivering toxic metabolites.[14] Nutritional immunity is well-characterized in the *Salmonella*-host competition for iron,[15–18] and has recently expanded to encompass competition for other key trace metal ions, such as zinc[19–21] and manganese.[22,23] Here, we demonstrate that MCOLN2 deprives *S*. Typhi of magnesium (Mg^{2+}), playing a major role in Mg^{2+}-based nutritional immunity for *Salmonella* replicating inside human cells.

This is an open access article under the CC BY license (http://creativecommons.org/licenses/by/4.0/).

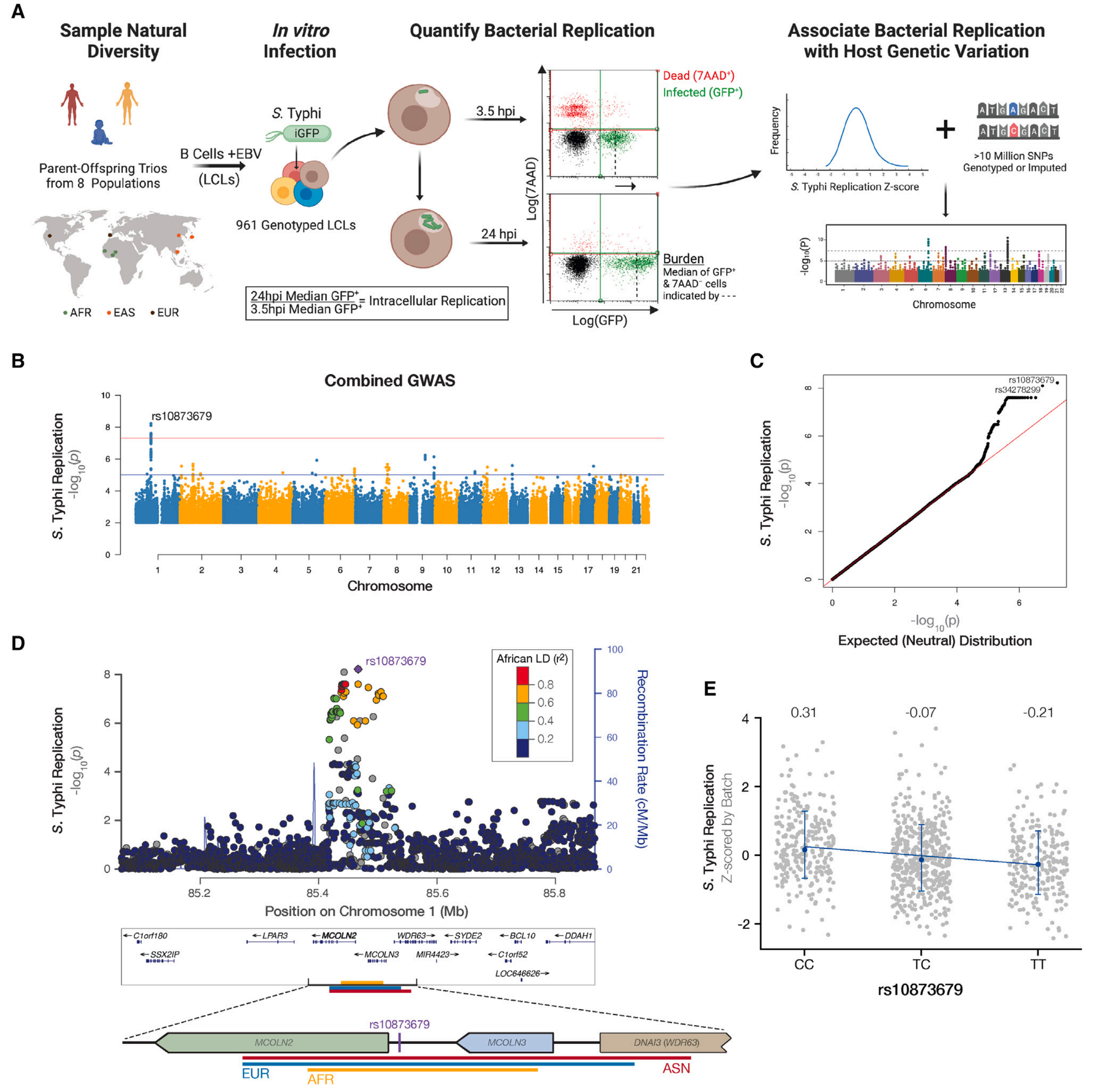

Figure 1. Cellular GWAS associates the rs10873679 locus with *S.* Typhi intracellular replication

(A) Hi-HoST cellular GWAS workflow. LCLs (Epstein-Barr virus [EBV]-immortalized B cells) were generated from eight populations sampled during the 1000 Genomes and HapMap Projects. The population locations are indicated on the map with dots color coded by continental ancestry. Green dots are three populations of African (AFR) ancestry: Esan in Nigeria (ESN), Gambians in the Western Division—Mandinka (GWD), and Yoruba in Ibadan, Nigeria (YRI). Orange dots are three populations of East Asian (EAS) ancestry: Kinh in Ho Chi Minh City, Vietnam (KHV); Han Chinese in Beijing, China (CHB); and Japanese in Tokyo, Japan (JPT). Brown dots are two populations of European (EUR) ancestry: Utah residents with northern and western European ancestry from the Center d'Etude du Polymorphisme Humain (CEPH) collection (CEU) and Iberian populations in Spain (IBS). Abbreviations used: iGFP, IPTG-inducible green fluorescent protein; hpi, hours post infection; 7AAD, 7-aminoactinomycin D cell viability stain; SNP, single-nucleotide polymorphism.

(B) Manhattan plot of cellular GWAS. p values were calculated using QFAM parents on the Z scored replication ratios (orange line, $p < 5 \times 10^{-8}$). The lead SNP on chromosome 1 is rs10873679 ($p = 6 \times 10^{-9}$).

(C) GWAS of *S.* Typhi intracellular replication has p values lower than expected from a neutral, χ^2, distribution (red line).

(D) A local Manhattan plot of the *S.* Typhi replication-associated locus on chromosome 1 (hg19 build) spans 400 kb up- and downstream of rs10873679. Dots for each SNP are color coded by African (AFR) linkage disequilibrium (LD) (r^2) from 1000 genomes Nov 2014 release. A 185 kb zoom in on the *MCOLN2/3* region

(legend continued on next page)

RESULTS

We identified common human single-nucleotide polymorphisms (SNPs) associated with *S*. Typhi intracellular replication, using Hi-HOST screening and family-based GWAS analysis[24] of 961 lymphoblastoid cell lines (LCLs) (EBV-immortalized B cells) from eight populations (Figure 1A; Table S1). LCLs are a powerful *in vitro* model because they are karyotypically normal, and B cells are a natural site of *Salmonella* replication *in vivo.*[25] Because intracellular replication, or host cell permissivity, is a demonstrated proxy for *Salmonella* virulence in whole organisms,[26] we used variable LCL permissivity to screen for human susceptibility or resistance factors. In this screen, we defined permissivity as the ratio of bacterial burden at 24 h to 3.5 h based on median green fluorescence intensity of live intracellular *S.* Typhi. This analysis revealed a single genome-wide significant locus (lead SNP is rs10873679, $p = 6 \times 10^{-9}$) on chromosome 1 (Figure 1B). A quantile-quantile plot demonstrated no overall inflation of the test statistic, with primarily rs10873679-linked SNPs deviating from the neutral distribution (Figure 1C). The association signal covers two genes in the mucolipin subfamily, *MCOLN2* and *MCOLN3* (Figure 1D). Mucolipins are a family of three inward rectifying divalent cation channels that localize to endolysosomal membranes and regulate vesicular trafficking.[27]

The minor (globally less common) C-allele of rs10873679 is associated with more intracellular *S*. Typhi replication (Figures 1E and S1). To link this to cellular physiology, we examined expression of *MCOLN2* and *MCOLN3* in RNA sequencing (RNA-seq) of 1000 Genomes LCLs.[28] The C-allele associated with less *MCOLN2* expression (Figure 2A; $p < 2 \times 10^{-16}$), while rs10873679 was not associated with a significant difference in *MCOLN3* expression (Figure 2B). In confirmation, the C-allele also associated with reduced MCOLN2 protein abundance in a quantitative mass spectrometry analysis of HapMap LCLs[29] (Figure 2C; $p = 0.01$). In this same analysis, MCOLN3 protein was only detected in 9 LCLs. This was insufficiently powered to draw a conclusion, although there was no evidence for association of MCOLN3 protein with rs10873679 genotype with these limited numbers. Together, the rs10873679 C-allele's association with both more *S*. Typhi replication and less MCOLN2 mRNA and protein suggested that MCOLN2 restricts *S*. Typhi intracellular replication.

Strengthening this model, *MCOLN2* is upregulated in human macrophages after treatment with M1 polarizing LPS and IFN-γ,[30] which indicates that *MCOLN2* is part of the host response. Similarly, we observed *MCOLN2* induction after *S*. Typhi infection (Figure 2D). If MCOLN2 is a restriction factor, we expected that ablating *MCOLN2* expression would increase intracellular *Salmonella* replication. Knocking down *MCOLN2*, but not *MCOLN3*, increased *S*. Typhi intracellular replication (Figure 2E), without affecting bacterial invasion or pyroptosis (Figure S2). This phenotype generalized to other human immune cells, as knocking down *MCOLN2* in THP-1 monocytes by RNAi (Figure 2F) or knocking out the gene using CRISPR-Cas (Figure 2G) resulted in an even greater increase in *S*. Typhi replication than in LCLs. In fact, *MCOLN2* knockout in THP-1s increased *S*. Typhi replication from 1- to 1.5-fold to 3- to 4-fold at 24 h, a large 2.5-fold increase in bacterial replication.

The rs10873679 locus was also associated with intracellular replication of *S*. Typhimurium ($p = 8.1 \times 10^{-7}$; Figure S3), a serovar used to model enteric fever in mice as *S.* Typhi is human-restricted; however, the impact of reducing *MCOLN2* expression is much smaller with *S.* Typhimurium (Figure 2H). This demonstrates that, while MCOLN2 is a key restriction factor for *S*. Typhi (knockout results in ∼150% more replication), it is an accessory factor for controlling *S*. Typhimurium (knockout results in ∼20% more replication). We confirmed lack of a large effect with *S.* Typhimurium by infecting susceptible C57BL/6J mice with *Mcoln2* knocked out[31] via intraperitoneal injection—which avoids restriction by stomach acid or variance introduced by gut microbiota—and quantified *S*. Typhimurium burden in the spleen 4 days post infection (Figure 2I). This revealed no significant difference in *S*. Typhimurium burden between *Mcoln2* genotypes, despite a modest trend of higher burden in $Mcoln2^{-/-}$ mice, which is not surprising given the small *in vitro* phenotype. This serovar difference could be explained by bacterial difference—only *S*. Typhi has the capacity to take advantage of a changed niche after MCOLN2's removal—or a differential host response, in which the more immunogenic *S*. Typhimurium induces additional restriction factors that prevent it from fully exploiting *MCOLN2* knockout. Regardless, our data demonstrate that MCOLN2 is a strong restriction factor for the human-specific serovar *S.* Typhi in cells, which underscores the value of cellular GWAS for identifying human-specific host-pathogens interactions.

To determine how MCOLN2 reduces *S*. Typhi replication, we used the intracellular bacteria as reporters of their own environment. We conducted transcriptomics at 16 h post infection (hpi), near maximum divergence of replication inside wild-type vs. $MCOLN2^{-/-}$ THP-1s and prior to restriction in wild-type THP-1s (Figures 3A and 3B). While >2,600 bacterial genes were detected, and expression of one-quarter of the bacterial transcriptome significantly changed between late-log inoculum and 16 hpi, differences between bacteria within wild-type and $MCOLN2^{-/-}$ cells were more modest with expression of no individual bacterial gene passing significance threshold after correction for multiple testing (Table S2). Therefore, we used gene set enrichment analysis to identify *S*. Typhi processes that were upregulated in *MCOLN2*-containing wild-type THP-1s. We generated a list of 15 gene sets of physiological processes associated with virulence or divalent cation transport (Figure 3C; Table S3). Only genes regulated by the PhoP/Q two-component system[32] were significantly enriched (NES = −1.81 with FDR q = 0.004) in bacteria living inside wild-type

highlights the lead SNP's location and indicates regions well linked ($r^2 > 0.6$) with rs10873679 by continental ancestry: African with a 71 kb orange bar; European (EUR) with a 121 kb blue bar; and Asian (ASN) with a 138 kb red bar.

(E) The rs10873679 C-allele is associated with increased *S*. Typhi replication. Means for each genotype are indicated above the scatterplots. Bars are ± SD. Regression slope ($\beta = -0.26 \pm 0.04$) is significantly less than zero ($p = 1.7 \times 10^{-9}$).

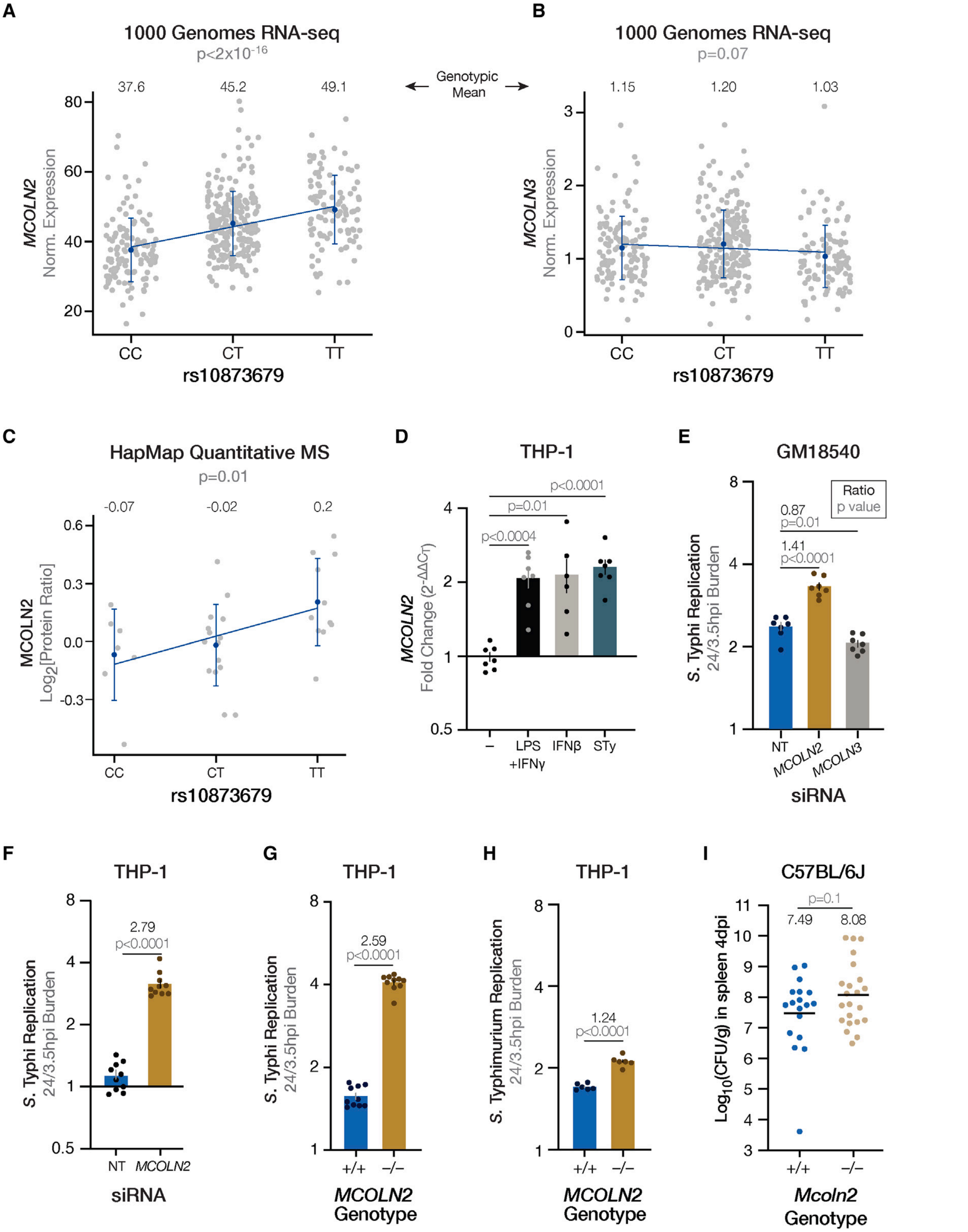

Figure 2. Increased mucolipin-2 expression restricts *S.* Typhi replication in human immune cell lines

(A) The rs10873679 C-allele associates with less *MCOLN2* mRNA expression in 1000 Genomes Project LCLs.[28] Linear regression of 448 LCLs (238 females and 210 males) is significant ($\beta = 5.8 \pm 0.6$; $p < 2 \times 10^{-16}$; adjusted $r^2 = 0.166$).

(legend continued on next page)

THP-1s compared with *MCOLN2*$^{-/-}$ THP-1s (Figure 3D). While *S.* Typhi within *MCOLN2*$^{-/-}$ cells upregulate PhoP/Q targets (10.7-fold more expression than late-log), induction is greater in bacteria inside wild-type cells (13.3-fold).

To determine if PhoP/Q signaling contributes to replication in *MCOLN2* knockout cells, we infected THP-1s with the Ty800 Δ*phoPQ* strain,[33] which revealed that most (~75%) of the increased replication inside *MCOLN2*$^{-/-}$ requires intact PhoPQ signaling (Figure 3E). Chief among PhoP/Q targets is the SPI-2 T3SS, which injects effectors to maintain *Salmonella*'s intracellular niche. Removing an essential component of the SPI-2 T3SS basal body (*ssaT*) to prevent any secretion caused no change in *S.* Typhi replication within wild-type THP-1s (compare blue bars in Figure 3F). This contrasts with *S.* Typhimurium[34,35] but is consistent with past *S.* Typhi literature.[36] In contrast, replication is reduced in *MCOLN2*$^{-/-}$ cells, suggesting that roughly half of the PhoP/Q-dependent increase in *S.* Typhi replication depends on SPI-2 effectors (Figure 3F). This indicates the SPI-2 independence of *S.* Typhi replication in THP-1 monocytes is actually an MCOLN2-dependent host response that suppresses the fitness advantage provided by *S.* Typhi's SPI-2 effectors.

Our results demonstrate that *S.* Typhi replicating inside *MCOLN2*$^{-/-}$ monocytes upregulate PhoP targets, which significantly boosts replication. However, in wild-type cells, the even greater induction of PhoP targets is not sufficient to increase replication, so we speculated that the PhoP upregulation was a symptom of a restrictive condition enhanced by MCOLN2. Three potentially restricting conditions in the SCV lead to more PhoP activity: PhoP/Q is repressed by high Mg^{2+}[37] and activated by cationic antimicrobial peptides[38] or acidification.[39,40] Since MCOLN2 is a divalent cation channel, PhoP/Q was most likely responding to reduced Mg^{2+} concentrations, which, along with Zn^{2+}, are limited in SCVs.[41,42] Indeed, the PhoP-activated Mg^{2+} importers *mgtA* and *mgtB* were both upregulated more in bacteria inside wild-type THP-1s (Figures 3D and S4). Therefore, the transcriptomics and *phoPQ* mutant infection data suggested a simple hypothesis: MCOLN2 deprives *S.* Typhi of Mg^{2+}. To test this, we repleted Mg^{2+} 2 h after infecting and measured bacterial replication (Figure 4A). Mg^{2+} supplementation disproportionately benefited bacterial replication inside wild-type THP-1s (1.6-fold in wild-type vs. 1.2-fold in knockout THP-1s; interaction p = 0.002). Similar results were also observed with *S.* Typhimurium (Figure 4B). While our transcriptomics could also support a role for Zn^{2+}, zinc repletion did not have interactions with the *MCOLN2* genotype, meaning it was similarly toxic to *S.* Typhi inside both *MCOLN2*$^{-/-}$ and wild-type THP-1 cells (Figure 4C; interaction p = 0.3). This agrees with previous findings that high concentrations of zinc are toxic to *Salmonella*.[43] However, *S.* Typhi inside *MCOLN2* knockout cells are not more susceptible to Zn^{2+} repletion, so we infer that MCOLN2 does not help *S.* Typhi resist zinc toxicity. Together, these data demonstrate that intracellular replication is held back by magnesium starvation and not zinc toxicity.

This Mg^{2+} starvation model is supported by whole-endolysosome patch-clamp measurements. While previous studies using whole-cell patch-clamping have demonstrated that MCOLN1 is permeable to most monovalent and divalent cations,[44] there has been no direct evidence showing that MCOLN2 conducts Mg^{2+} from the lumen of endolysosomes into the cytosol. To determine if human MCOLN2 can conduct Mg^{2+}, it was expressed in HEK293 cells, and endolysosomal organelles were isolated for direct patch-clamping using a previously established approach.[45–47] While no significant Mg^{2+} currents were seen in non-transfected endolysosomes (Figure 4D), application of an MCOLN2-specific small-molecule agonist, ML2-SA1,[46] evoked inward Mg^{2+} currents on intact endolysosomes isolated from MCOLN2-expressing cells (Figure 4E). We also observed Mg^{2+} currents after administration of $PI(3,5)P_2$, a putative endogenous agonist[48] (Figure 4F). This is especially intriguing in light of *Salmonella*'s known manipulation of phosphoinositides through the sopB effector[49,50] and our previous finding that a host protein that regulates $PI(3,5)P_2$ is associated with *Salmonella* invasion and typhoid fever risk.[7] These results demonstrate that MCOLN2 conducts Mg^{2+} and is capable of serving as a channel for Mg^{2+} out of endolysosomes and into the cytosol.

(B) The rs10873679 genotype does not associate with *MCOLN3* mRNA expression in the same dataset ($\beta = -0.05 \pm 0.03$; p = 0.07; adjusted $r^2 = 0.005$). LCLs in (A) and (B) are from four European populations (91 CEU, 92 FIN, 86 GBR, and 91 TSI) and one African population (88 YRI) and consist of 124 CC, 217 TC, and 107 TT individuals.

(C) The rs10873679 C-allele associates with less MCOLN2 protein expression in 33 LCLs (19 female and 14 male) measured with quantitative mass spectrometry.[29] LCLs are from four populations: 18 CEU, 10 YRI, 4 CHB, and 1 JPT. Linear regression is significant for MCOLN2 ($\beta = 0.15 \pm 0.05$, p = 0.01; adjusted $r^2 = 0.017$). In (A)–(C), bars are mean ± SD.

(D) Both interferon treatment and *S.* Typhi (STy) infection (MOI 10 for 24 h) stimulate *MCOLN2* expression in THP-1s. Expression measured by qRT-PCR and quantified by $\Delta\Delta C_T$ (ΔC_T stimulated – ΔC_T untreated). Seven replicates from three experiments. p values are from Dunnett's T3 comparison after Welch's ANOVA (p < 0.0001).

(E) In the LCL GM18540 (derived from a female CHB), *MCOLN2* knockdown increases *S.* Typhi replication, while *MCOLN3* knockdown modestly decreases *S.* Typhi replication in comparison with non-targeting (NT) siRNA. Seven replicates from three experiments. Knockdown qPCR: 0.33-fold (±0.14) of NT *MCOLN2* expression and 0.53-fold (±0.04) of NT *MCOLN3* expression. p values are from Dunnett post-hoc comparison with NT following a one-way ANOVA (main effect p < 0.0001).

(F) *MCOLN2* knockdown (0.09-fold [±0.02] of NT) increases *S.* Typhi replication in THP-1s. Ten replicates from two experiments.

(G) CRISPR-Cas9 knockout of *MCOLN2* increases *S.* Typhi replication in THP-1s. Ten replicates from two experiments.

(H) *S.* Typhimurium has a minor growth advantage in *MCOLN2* knockout THP-1s. Six replicates from two experiments. In (E)–(H), ratios are mean in siRNA-treated/NT or knockout/wild-type cells. In (D)–(H), bars are mean ± SEM and all statistics are calculated with $\log_2$-transformed data.

(I) *Mcoln2* knockout does not significantly increase burden in C57BL/6J mice spleens 4 days post i.p. infection with 1,000 CFUs of late-log *S.* Typhimurium (14028s) tagged with p67GFP3.1. Eighteen wild types (10 female and 8 male) and 22 knockouts (13 female and 9 male) from six experiments. Lines are geometric means and $\log_{10}$(geo. mean) is shown above each genotype. p value calculated with $\log_{10}$-transformed data. Without the low outlier ($\log_{10}$[CFU] = 3.6; identified at Grubbs' $\alpha = 0.01$), the *Mcoln2*$^{+/+}$ $\log_{10}$(geo. mean) is 7.72 and p = 0.2. In (E)–(I), p values are from Welch's t test.

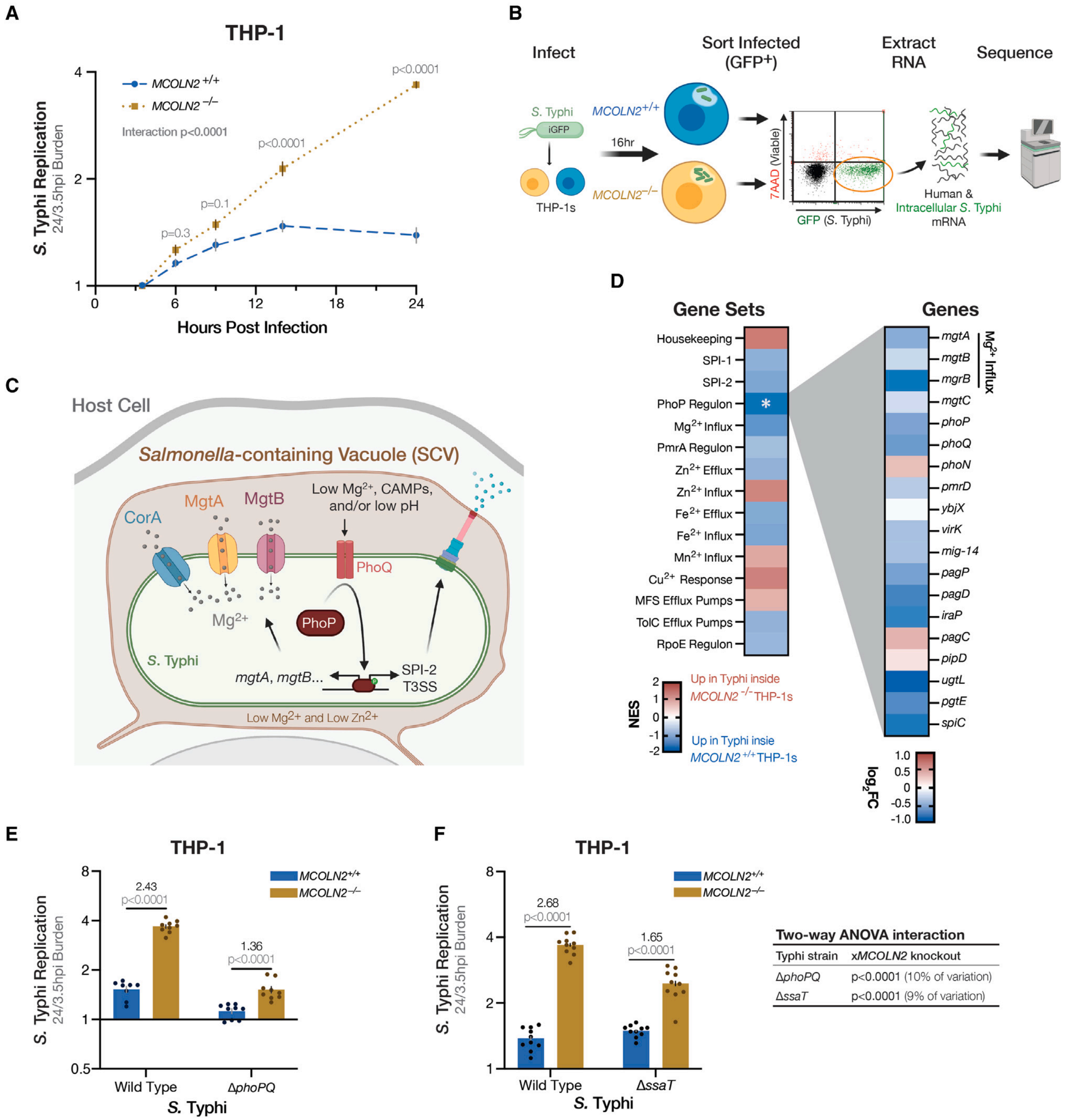

Figure 3. Intracellular *S.* Typhi replication inside *MCOLN2*$^{-/-}$ THP-1s depends on PhoP/Q

(A) *MCOLN2* knockout leads to faster *S.* Typhi replication inside THP-1s. Ten replicates from three experiments, except 6 hpi is six replicates from two experiments. *MCOLN2* genotype, hpi, and their interaction are all significant sources of variation ($p < 0.0001$) in a repeated measures ANOVA. Time point p values are from Šídák's post-hoc comparison of *MCOLN2*$^{+/+}$ and *MCOLN2*$^{-/-}$.

(B) Workflow used to sequence mRNA from intracellular *S.* Typhi 16 hpi in wild-type and knockout THP-1s

(C) Diagram of *S.* Typhi's PhoPQ-induced Mg^{2+} importers.

(D) RNA-seq of intracellular *S.* Typhi indicates that PhoP targets are upregulated more when MCOLN2 is present. Left: normalized enrichment score (NES) from gene set enrichment analysis of virulence- or cation-associated *S.* Typhi gene sets. Significant gene set (FDR $q < 0.05$) indicated by asterisk. Right: the $\log_2$(KO/WT expression) of PhoPQ regulon genes is plotted. Sixteen of 19 genes have a negative fold change (FC), indicating higher expression in WT.

(E) *PhoPQ* is required for most of the increase in intracellular replication observed with *MCOLN2*$^{-/-}$ THP-1s. Nine replicates from two experiments.

(legend continued on next page)

The repletion and electrophysiological evidence is further bolstered by genetic interaction of *MCOLN2* with *Salmonella* Mg^{2+} transporters. The importance of Mg^{2+} acquisition for *Salmonella* replication is underscored by its trio of Mg^{2+} uptake proteins: one constitutive, CorA, and two inducible, MgtA and MgtB. If knocking out *MCOLN2* increases Mg^{2+} availability, we theorized that these transporters would be necessary to uptake that extra Mg^{2+} and therefore essential for the enhanced replication inside *MCOLN2*$^{-/-}$ host cells. To test this, we generated a double knockout (Δ*mgtA*Δ*mgtB*), which lacks the high-affinity Mg^{2+} importers used in low-Mg^{2+} environments, like the $\leq 10\ \mu M$ concentration in the SCV.[41] In confirmation of our hypothesis, the double importer mutant is killed, instead of replicating, inside THP-1s (Figure 4G). Knocking out *MCOLN2* provides less of an advantage to the double importer mutant (increasing replication 60% in ΔΔ vs. 150% in wild-type *S*. Typhi; interaction $p < 0.001$). This corroborates the Mg^{2+} repletion and suggests that most of the enhanced replication in *MCOLN2*$^{-/-}$ THP-1s depends on increasing Mg^{2+} availability.

To test if this magnesium-MCOLN2 interaction occurs *in vivo*, we infected susceptible mice with a 1:1 ratio of double knockout (Δ*mgtA*Δ*mgtB* or ΔΔ) and wild-type *S.* Typhimurium by intraperitoneal injection. In theory, the increased Mg^{2+} availability in *Mcoln2*$^{-/-}$ mice would change the competitive index (CI) between double mutant and wild-type *S*. Typhimurium. As expected, Δ*mgtA*Δ*mgtB S*. Typhimurium is greatly attenuated compared with the wild type in C57BL/6J (CI = 0.024), and the double Mg^{2+}-importer mutant's attenuation is significantly more pronounced in *Mcoln2*$^{-/-}$ mice (CI = 0.007; Figure 4H). Based on our cellular findings, one would expect the reduced CI in *Mcoln2*$^{-/-}$ mice to be driven by more replication of the wild-type bacteria that can take advantage of increased Mg^{2+} availability in *Mcoln2*$^{-/-}$ mice; instead, we observed no significant change in wild-type bacteria replication in *Mcoln2*$^{-/-}$ mice ($\Delta\Delta_{in-/-}/\Delta\Delta_{in+/+} = 0.48$ with $p = 0.4$) accompanied with significantly less replication of double mutant bacteria in *Mcoln2*$^{-/-}$ mice ($\Delta\Delta_{in-/-}/\Delta\Delta_{in+/+} = 0.14$ with $p = 0.002$; Figure 4I). The genetic interaction of a magnesium importer mutant with murine host *Mcoln2* genotype ($p = 0.0002$) leads us to conclude that murine Mcoln2, like human MCOLN2, affects Mg^{2+} accessibility by *Salmonella* during infection. However, the comparative growth disadvantage of the *S*. Typhimurium double importer mutant in *Mcoln2* knockout mice contrasting with the comparative growth advantage of wild-type *S.* Typhi in *MCOLN2*$^{+/+}$ human THP-1 cells (see Figure 4G) suggests that mucolipin-2's impact on Mg^{2+} availability during infection depends on context, likely including *Salmonella* serovar and host species as well as the infected cell type or tissue. Despite these differences, the *in vivo* and *in vitro* data concur that mucolipin-2 changes Salmonellae replication by altering their access to Mg^{2+}.

In the simplest version of our model, removing human MCOLN2 increases Mg^{2+} availability to *S.* Typhi, which relieves a nutrient limitation and directly increases bacterial replication. However, ~1/3 of the increased bacterial replication inside *MCOLN2* knockout cells is not explained by manipulating Mg^{2+} availability or uptake. We theorized that this putatively Mg^{2+}-independent replication boost in *MCOLN2*$^{-/-}$ cells could still be PhoP regulated, as we had already identified other PhoP-targets, namely SPI-2 T3SS, which further benefit *S*. Typhi replication when *MCOLN2* is knocked out. To test this, we repleted Mg^{2+} after infecting THP-1s with *S.* Typhi Δ*phoPQ* (Figure 4J). Mg^{2+} increased replication of Δ*phoPQ* bacteria, as it partially overcomes the inability to fully upregulate *mgtA* and *mgtB*. Furthermore, the combined Mg^{2+} repletion and *phoPQ* deletion removed any discernable difference in *S*. Typhi replication between *MCOLN2* genotypes. Thus, enhanced bacterial replication in the absence of *MCOLN2* depends on both Mg^{2+}-independent effects of PhoPQ and PhoPQ-independent effects of Mg^{2+} availability (Figure 4K).

DISCUSSION

In this report, we directly connect expression of the divalent cation channel MCOLN2 with variable immune cell permissivity to *S*. Typhi. For *S*. Typhimurium, intracellular replication regulates outcomes in mouse models of enteric fever,[26,51] and, therefore, *S.* Typhi replication likely also correlates with disease outcome in humans. Unfortunately, there is no published GWAS of typhoid severity or clinical outcome and only one study on typhoid fever onset, which identified an association between the MHC region and susceptibility.[52] Thus, determining the clinical significance of rs10873679 in humans awaits well-powered studies for this disease phenotype. Our findings also underscore that, despite great insights gleaned from mouse models of *S.* Typhimurium infection, studies of genetic diversity using human-specific pathogens in human cells provide unique insight.

Furthermore, we showed that *MCOLN2* ablation reduced the low-Mg^{2+} stress faced by intracellular *S*. Typhi based on lowered expression of Mg^{2+}-regulated PhoP targets (including key Mg^{2+} transporters) and reduced benefit of Mg^{2+} repletion. Thus, the divalent cation channel MCOLN2 exerts restriction pressure on *S*. Typhi inside human monocytes by reducing Mg^{2+} availability, which is similar to how the divalent cation transporter Slc11a1 (Nramp1) is proposed to restrict *S.* Typhimurium inside murine macrophages.[53] It is worth noting that C57BL/6J mice are highly susceptible to *Salmonella* due to a deleterious mutation in *Slc11a1*, which means divalent cation transport in their immune cells is already disrupted in a way that advantages *S*. Typhimurium replication.[54] It is possible that future work will find a greater or different effect of Mcoln2 in mice with functional Slc11a1. Despite the similarity of proposed mechanisms for the effects of MCOLN2 and Slc11a1, transport of Mg^{2+} by Slc11a1 has never been demonstrated nor has human SLC11A1 ever been shown to restrict *Salmonella* replication. This underscores the importance of our discovery that MCOLN2 is a bona fide Mg^{2+} channel between endolysosomes and the cytosol, as it bolsters our genetic and functional evidence of Mg^{2+}-based nutritional immunity

(F) *S*. Typhi Δ*ssaT* has no effect on intracellular replication in WT THP-1s and partially accounts for the requirement of *phoPQ* to achieve maximal replication in *MCOLN2*$^{-/-}$ THP-1s. Ten replicates from three experiments. p values in (E) and (F) are from Šídák's comparison of *MCOLN2*$^{+/+}$ to *MCOLN2*$^{-/-}$ following two-way ANOVAs finding significant main effects and interaction (all $p < 0.0001$). Statistics in (A), (E), and (F) use $\log_2$-transformed replication ratios. Bars in (A), (E), and (F) are mean ± SEM.

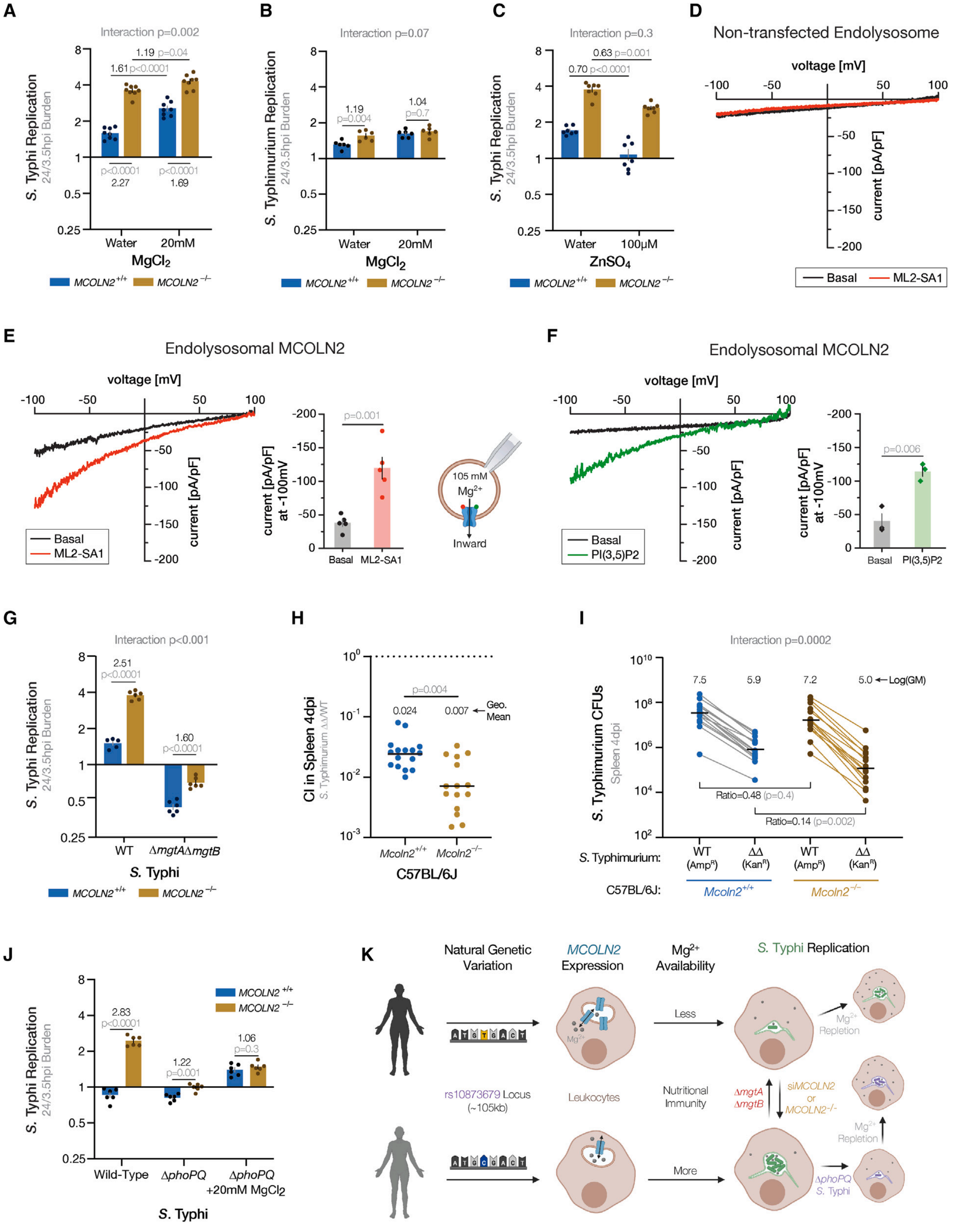

(legend on next page)

against intracellular *Salmonella*. Thus, our multi-disciplinary approach to understanding human variation, which revealed the first common human genetic difference that regulates intracellular resistance to *Salmonella*, has also led to the identification of the critical host factor that restricts *S.* Typhi by Mg^{2+} deprivation.

Identifying human MCOLN2 as a host factor that drastically reduces *Salmonella* replication by lowering Mg^{2+} availability highlights the key role played by Mg^{2+} in nutritional immunity. This builds on a line of work identifying the sophisticated regulatory network in *S.* Typhimurium that allows it to respond to the low-Mg^{2+} environment of the SCV.[37,55] Notably, these investigations into *Salmonella* response to low Mg^{2+} have been conducted with non-typhoidal *S*. Typhimurium. While much of this regulatory system is likely preserved in *S*. Typhi, the much greater sensitivity of *S*. Typhi to MCOLN2 ablation suggests that some component of this low Mg^{2+} response is not conserved between the serovars. Future studies investigating this difference could reveal key serovar-specific virulence strategies.

Our finding that MCOLN2 restricts *S*. Typhi also explains why it is an ISG, despite previous findings that it increases macrophage susceptibility to endocytosed viruses including influenza A virus (Orthomyxoviridae) and yellow fever virus (Flaviviridae).[56] The induction of MCOLN2 expression in activated immune cells therefore provides two mechanisms whereby this channel could regulate infection—Ca^{2+} currents regulating endocytic events and Mg^{2+} currents affecting Mg^{2+} acquisition. This identifies the *MCOLN2* locus as a possible site of balancing selection between different infectious disease pressures—viruses that use the endocytic pathway for entry might select for people with less *MCOLN2* expression, while Salmonellae infections might select for people with more *MCOLN2* expression. This balancing selection could explain the wide distribution of both rs10873679 alleles in populations around the world, and, ultimately, it highlights the persistent and complex power of infectious disease as an evolutionary pressure shaping human evolution.

Limitations of the study

Our genetic association work in this study is limited to LCLs. Therefore, the association of rs10873679 with *S.* Typhi replication will need to be examined in other cell types, with varying immune cell polarization, and ultimately in human populations. Similarly, the functional studies of *MCOLN2* were consistent in LCLs and THP-1 monocytes but have not been extended to other cell types. The MCOLN2 patch-clamp experiments were conducted using overexpression in HEK293 cells, and there may be differences with endogenous expression in immune cells. As noted above, the effects of *MCOLN2* varies across different *Salmonella enteria* serovars, and future studies will need to define the mechanistic underpinnings of these differences.

STAR★METHODS

Detailed methods are provided in the online version of this paper and include the following:

Figure 4. MCOLN2 reduces *Salmonella* replication by reducing magnesium availability

(A) Mg^{2+} supplementation partially rescues *S*. Typhi replication in *MCOLN2*$^{-/-}$ THP-1s. Eight replicates from two experiments. Mg^{2+} supplementation and *MCOLN2* genotype are both significant ($p < 0.0001$) in two-way ANOVA. Bar plots throughout figure are mean ± SEM.

(B) Mg^{2+} supplementation overcomes the slight restriction of *S*. Typhimurium by MCOLN2 in THP-1 monocytes. Six replicates from two experiments. Both supplementation ($p = 0.0004$) and *MCOLN2* genotype ($p = 0.006$) are significant sources of variation by two-way ANOVA.

(C) Zn^{2+} supplementation reduces *S*. Typhi replication in THP-1 monocytes independently from *MCOLN2* genotype. Seven replicates from two experiments. In a two-way ANOVA, Zn^{2+} supplementation and *MCOLN2* genotype are significant sources of variation ($p < 0.0001$), but their interaction is not ($p = 0.3$). In (A)–(C), mock is the addition of an equal volume filter-sterilized DI water and p values comparing means are from Tukey's post-hoc multiple comparison tests.

(D) No significant ML2-SA1-evoked Mg^{2+} currents were recorded from endolysosomes isolated from non-transfected HEK293 cells. Representative I-V traces of human MCOLN2-mediated whole-endolysosome Mg^{2+} currents before (black, basal) and after (red) treatment with the MCOLN2 small-molecule agonist ML2-SA1.

(E) MCOLN2 conducts Mg^{2+} in endolysosomes when treated with ML2-SA1. A representative recording of Mg^{2+} currents from isolated endolysosomes expressing human MCOLN2 is shown. Bar plots show mean values ± SEM at −100 mV from independent experiments. p values from paired t test. The cartoon of whole-endolysosome patch-clamp configuration indicates the direction of the inward (flow out of the vesicles) Mg^{2+} currents mediated by MCOLN2.

(F) Natural MCOLN2 agonist, PI(3,5)P2, also elicits Mg^{2+} currents through MCOLN2. Representative Mg^{2+} currents from endolysosomes isolated from MCOLN2 transfected HEK293 cells, basal (black) or elicited by an application of 10 μM PI(3,5)P2 (green). Statistical summary same as (E).

(G) PhoPQ-induced magnesium importers MgtA and MgtB are required for half the *S*. Typhi replication benefit in *MCOLN2*$^{-/-}$ THP-1s. Six replicates from two experiments. In a two-way ANOVA, *MCOLN2* genotype, *mgtA*/*mgtB* deletion, and their interaction are all significant sources of variation ($p < 0.0001$). In (B), (C), and (G), p values comparing two means are from post-hoc Šidák's multiple comparison tests.

(H) The fitness disadvantage of an *S*. Typhimurium Δ*mgtA*Δ*mgtB* mutant (ΔΔ) competing against wild-type (WT) *S*. Typhimurium is enhanced when competing in *Mcoln2*$^{-/-}$ mice. Spleens harvested 4 dpi with 1,000 CFUs of each WT and ΔΔ *S*. Typhimurium inoculum. Lines indicate geometric means (GM) of competitive indices (CI) and shown above each mouse genotype. One outlier (CI = 1.21) in *Mcoln2*$^{+/+}$ was identified by a Grubbs' test ($\alpha = 0.0001$) and removed. p value from Welch's t test. Outlier test and t test calculated on $\log_{10}$(CFUs). Sixteen *Mcoln2*$^{+/+}$ mice (10 female and 6 male) and 15 *Mcoln2*$^{-/-}$ mice (4 female and 11 male) from 4 experiments.

(I) The enhanced CI in *Mcoln2*$^{-/-}$ mice is driven by ΔΔ *S*. Typhimurium replicating less inside *Mcoln2*$^{-/-}$ than *Mcoln2*$^{+/+}$ mice. Data are from the same experiments and mice as in (H) and exclude the same outlier. Diagonal lines connect CFU counts from the same mouse while horizontal black lines indicate the $\log_{10}$-geometric mean (GM) of each group. Ratios are GM in *Mcoln2*$^{-/-}$ mice over GM in *Mcoln2*$^{+/+}$ mice. Interaction p value is from repeated measure two-way ANOVA in which CFUs from the same mouse are paired. Mouse *Mcoln2* genotype ($p = 0.02$), bacteria *mgtA/B* genotype ($p < 0.0001$), and their interaction ($p = 0.0002$) are all significant sources of variation. p values comparing conditions are from post-hoc Šídák's multiple comparison tests. All statistics calculated using $\log_{10}$[CFUs].

(J) *MCOLN2* knockout does not benefit Δ*phoPQ S*. Typhi replication inside THP-1s when Mg^{2+} is repleted. Six replicates from two experiments. In a three-way ANOVA, $MgCl_2$ treatment, *phoPQ* deletion, *MCOLN2* genotype, and all two-way interactions are significant ($p < 0.0001$). p values comparing two means are from post-hoc Welch's t tests.

(K) Model of natural human genetic variation altering intracellular *S*. Typhi replication.

- KEY RESOURCES TABLE
- RESOURCE AVAILABILITY
  - Lead contact
  - Materials availability
  - Data and code availability
- EXPERIMENTAL MODEL AND SUBJECT DETAILS
  - Human cells
  - Mice
  - Bacteria
- METHOD DETAILS
  - Infection (gentamicin-protection) assays
  - Cellular GWAS
  - Human gene expression analyses
  - RNAi experiments and knockdown confirmation
  - Inducing and measuring *MCOLN2* expression
  - Mouse infections
  - Fluorescence-activated cell sorting (FACS)
  - *S.* Typhi infected THP-1 RNA extraction
  - THP-1 and intracellular *S.* Typhi Dual RNA-seq
  - *S.* Typhi gene set enrichment analysis (GSEA)
  - Endolysosomal patch-clamp experiments
- QUANTIFICATION AND STATISTICAL ANALYSIS

SUPPLEMENTAL INFORMATION

Supplemental information can be found online at https://doi.org/10.1016/j.xgen.2023.100290.

ACKNOWLEDGMENTS

We thank the Duke University School of Medicine Sequencing and Genomic Technologies Shared Resource for providing services. We thank Kristin Cleveland and Duke DLAR Breeding Core personnel for breeding and maintenance of mouse lines. We thank the investigators and individuals from diverse populations genotyped as part of the 1000 Genomes and HapMap Projects who have made their LCLs available through the Coriell Institute. We thank Marco Keller, Franz Bracher, and Christian Grimm (LMU Munich, Germany) for providing ML2-SA1 and human MCOLN2 vector. We thank Samuel I. Miller and members of the Ko lab for useful discussion. D.C.K., K.D.G., L.W., C.E.A., J.S.B., Y.C., and M.R.G. were supported by NIH R01AI118903. K.D.G. was supported by NIH F31AI136313. J.S.B. was supported by NIH F31AI143147. Z.Y. and M.B. were supported by the Deutsche Forschungsgemeinschaft (DFG, German Research Foundation) TRR152. R.P. was funded by the NHLBI Division of Intramural Research (ZIA HL006075). C.-C.C. was supported by the National Science and Technology Council (R.O.C. Taiwan), MOST 110-2320-B-002-022 (National Taiwan University), NTU-112L7818, and the National Health Research Institutes, Taiwan (NHRI-EX112-11119SC). All schematic images were generated using Biorender.com and figures were made with Adobe Illustrator v.27.

AUTHOR CONTRIBUTIONS

Conceptualization, K.D.G., L.W., J.S.B., Y.C., and D.C.K.; formal analysis, K.D.G., L.W., and C.-C.C.; investigation, K.D.G., L.W., Z.Y., C.E.A., J.S.B., Y.C., M.R.G., C.-C.C., and D.C.K.; funding acquisition, K.D.G. and D.C.K.; supervision, M.B., C.-C.C., and D.C.K.; resources, K.D.G., C.E.A., J.S.B., F.B., M.B., R.P., C.-C.C., and D.C.K.; writing – original draft, K.D.G. and D.C.K.; writing – review & editing, K.D.G., J.S.B., C.-C.C., and D.C.K.

DECLARATION OF INTERESTS

The authors declare no competing interests.

INCLUSION AND DIVERSITY

We worked to ensure diversity in experimental samples through the selection of the cell lines. We support inclusive, diverse, and equitable conduct of research.

Received: November 2, 2022
Revised: January 24, 2023
Accepted: February 27, 2023
Published: April 4, 2023

REFERENCES

1. Gibbs, K.D., Schott, B.H., and Ko, D.C. (2022). The awesome power of human genetics of infectious disease. Annu. Rev. Genet. *56*, 41–62. https://doi.org/10.1146/annurev-genet-080320-010449.
2. Barreiro, L.B., Tailleux, L., Pai, A.A., Gicquel, B., Marioni, J.C., and Gilad, Y. (2012). Deciphering the genetic architecture of variation in the immune response to Mycobacterium tuberculosis infection. Proc. Natl. Acad. Sci. USA *109*, 1204–1209. https://doi.org/10.1073/pnas.1115761109.
3. Fairfax, B.P., Humburg, P., Makino, S., Naranbhai, V., Wong, D., Lau, E., Jostins, L., Plant, K., Andrews, R., McGee, C., and Knight, J.C. (2014). Innate immune activity conditions the effect of regulatory variants upon monocyte gene expression. Science *343*, 1246949. https://doi.org/10.1126/science.1246949.
4. Lee, M.N., Ye, C., Villani, A.C., Raj, T., Li, W., Eisenhaure, T.M., Imboywa, S.H., Chipendo, P.I., Ran, F.A., Slowikowski, K., et al. (2014). Common genetic variants modulate pathogen-sensing responses in human dendritic cells. Science *343*, 1246980. https://doi.org/10.1126/science.1246980.
5. Ko, D.C., Shukla, K.P., Fong, C., Wasnick, M., Brittnacher, M.J., Wurfel, M.M., Holden, T.D., O'Keefe, G.E., Van Yserloo, B., Akey, J.M., and Miller, S.I. (2009). A genome-wide in vitro bacterial-infection screen reveals human variation in the host response associated with inflammatory disease. Am. J. Hum. Genet. *85*, 214–227. https://doi.org/10.1016/j.ajhg.2009.07.012.
6. Wang, L., Pittman, K.J., Barker, J.R., Salinas, R.E., Stanaway, I.B., Williams, G.D., Carroll, R.J., Balmat, T., Ingham, A., Gopalakrishnan, A.M., et al. (2018). An atlas of genetic variation linking pathogen-induced cellular traits to human disease. Cell Host Microbe *24*, 308–323.e6. https://doi.org/10.1016/j.chom.2018.07.007.
7. Alvarez, M.I., Glover, L.C., Luo, P., Wang, L., Theusch, E., Oehlers, S.H., Walton, E.M., Tram, T.T.B., Kuang, Y.L., Rotter, J.I., et al. (2017). Human genetic variation in VAC14 regulates Salmonella invasion and typhoid fever through modulation of cholesterol. Proc. Natl. Acad. Sci. USA *114*, E7746–E7755. https://doi.org/10.1073/pnas.1706070114.
8. Bourgeois, J.S., Wang, L., Rabino, A.F., Everitt, J., Alvarez, M.I., Awadia, S., Wittchen, E.S., Garcia-Mata, R., and Ko, D.C. (2021). ARHGEF26 enhances Salmonella invasion and inflammation in cells and mice. PLoS Pathog. *17*, e1009713. https://doi.org/10.1371/journal.ppat.1009713.
9. Johnson, R., Mylona, E., and Frankel, G. (2018). Typhoidal Salmonella: distinctive virulence factors and pathogenesis. Cell Microbiol. *20*, e12939. https://doi.org/10.1111/cmi.12939.
10. Schoggins, J.W., MacDuff, D.A., Imanaka, N., Gainey, M.D., Shrestha, B., Eitson, J.L., Mar, K.B., Richardson, R.B., Ratushny, A.V., Litvak, V., et al. (2014). Pan-viral specificity of IFN-induced genes reveals new roles for cGAS in innate immunity. Nature *505*, 691–695.
11. Hood, M.I., and Skaar, E.P. (2012). Nutritional immunity: transition metals at the pathogen-host interface. Nat. Rev. Microbiol. *10*, 525–537. https://doi.org/10.1038/nrmicro2836.
12. Núñez, G., Sakamoto, K., and Soares, M.P. (2018). Innate nutritional immunity. J. Immunol. *201*, 11–18. https://doi.org/10.4049/jimmunol.1800325.

13. Palmer, L.D., and Skaar, E.P. (2016). Transition metals and virulence in bacteria. Annu. Rev. Genet. *50*, 67–91. https://doi.org/10.1146/annurev-genet-120215-035146.
14. Chen, M., Sun, H., Boot, M., Shao, L., Chang, S.J., Wang, W., Lam, T.T., Lara-Tejero, M., Rego, E.H., and Galán, J.E. (2020). Itaconate is an effector of a Rab GTPase cell-autonomous host defense pathway against Salmonella. Science *369*, 450–455. https://doi.org/10.1126/science.aaz1333.
15. Cassat, J.E., and Skaar, E.P. (2013). Iron in infection and immunity. Cell Host Microbe *13*, 509–519. https://doi.org/10.1016/j.chom.2013.04.010.
16. Nairz, M., Ferring-Appel, D., Casarrubea, D., Sonnweber, T., Viatte, L., Schroll, A., Haschka, D., Fang, F.C., Hentze, M.W., Weiss, G., and Galy, B. (2015). Iron regulatory proteins mediate host resistance to Salmonella infection. Cell Host Microbe *18*, 254–261. https://doi.org/10.1016/j.chom.2015.06.017.
17. Nairz, M., Fritsche, G., Brunner, P., Talasz, H., Hantke, K., and Weiss, G. (2008). Interferon-gamma limits the availability of iron for intramacrophage Salmonella typhimurium. Eur. J. Immunol. *38*, 1923–1936. https://doi.org/10.1002/eji.200738056.
18. Weinberg, E.D. (1975). Nutritional immunity. Host's attempt to withold iron from microbial invaders. JAMA *231*, 39–41. https://doi.org/10.1001/jama.231.1.39.
19. Gammoh, N.Z., and Rink, L. (2017). Zinc in infection and inflammation. Nutrients *9*, 624. https://doi.org/10.3390/nu9060624.
20. Cerasi, M., Liu, J.Z., Ammendola, S., Poe, A.J., Petrarca, P., Pesciaroli, M., Pasquali, P., Raffatellu, M., and Battistoni, A. (2014). The ZupT transporter plays an important role in zinc homeostasis and contributes to Salmonella enterica virulence. Metallomics *6*, 845–853.
21. Ammendola, S., Pasquali, P., Pistoia, C., Petrucci, P., Petrarca, P., Rotilio, G., and Battistoni, A. (2007). High-affinity Zn2+ uptake system ZnuABC is required for bacterial zinc homeostasis in intracellular environments and contributes to the virulence of Salmonella enterica. Infect. Immun. *75*, 5867–5876.
22. Kehl-Fie, T.E., and Skaar, E.P. (2010). Nutritional immunity beyond iron: a role for manganese and zinc. Curr. Opin. Chem. Biol. *14*, 218–224. https://doi.org/10.1016/j.cbpa.2009.11.008.
23. Papp-Wallace, K.M., and Maguire, M.E. (2006). Manganese transport and the role of manganese in virulence. Annu. Rev. Microbiol. *60*, 187–209.
24. Purcell, S., Sham, P., and Daly, M.J. (2005). Parental phenotypes in family-based association analysis. Am. J. Hum. Genet. *76*, 249–259. https://doi.org/10.1086/427886.
25. García-Gil, A., Lopez-Bailon, L.U., and Ortiz-Navarrete, V. (2019). Beyond the antibody: B cells as a target for bacterial infection. J. Leukoc. Biol. *105*, 905–913. https://doi.org/10.1002/JLB.MR0618-225R.
26. Fields, P.I., Swanson, R.V., Haidaris, C.G., and Heffron, F. (1986). Mutants of *Salmonella* Typhimurium that cannot survive within the macrophage are avirulent. Proc. Natl. Acad. Sci. USA *83*, 5189–5193.
27. Spix, B., Chao, Y.K., Abrahamian, C., Chen, C.C., and Grimm, C. (2020). TRPML cation channels in inflammation and immunity. Front. Immunol. *11*, 225. https://doi.org/10.3389/fimmu.2020.00225.
28. Lappalainen, T., Sammeth, M., Friedländer, M.R., 't Hoen, P.A.C., Monlong, J., Rivas, M.A., Gonzàlez-Porta, M., Kurbatova, N., Griebel, T., Ferreira, P.G., et al. (2013). Transcriptome and genome sequencing uncovers functional variation in humans. Nature *501*, 506–511. https://doi.org/10.1038/nature12531.
29. Wu, L., Candille, S.I., Choi, Y., Xie, D., Jiang, L., Li-Pook-Than, J., Tang, H., and Snyder, M. (2013). Variation and genetic control of protein abundance in humans. Nature *499*, 79–82. https://doi.org/10.1038/nature12223.
30. Martinez, F.O., Gordon, S., Locati, M., and Mantovani, A. (2006). Transcriptional profiling of the human monocyte-to-macrophage differentiation and polarization: new molecules and patterns of gene expression. J. Immunol. *177*, 7303–7311.
31. Sun, L., Hua, Y., Vergarajauregui, S., Diab, H.I., and Puertollano, R. (2015). Novel role of TRPML2 in the regulation of the innate immune response. J. Immunol. *195*, 4922–4932. https://doi.org/10.4049/jimmunol.1500163.
32. Zwir, I., Latifi, T., Perez, J.C., Huang, H., and Groisman, E.A. (2012). The promoter architectural landscape of the Salmonella PhoP regulon. Mol. Microbiol. *84*, 463–485. https://doi.org/10.1111/j.1365-2958.2012.08036.x.
33. Hohmann, E.L., Oletta, C.A., Killeen, K.P., and Miller, S.I. (1996). phoP/phoQ-deleted Salmonella typhi (Ty800) is a safe and immunogenic single-dose typhoid fever vaccine in volunteers. J. Infect. Dis. *173*, 1408–1414. https://doi.org/10.1093/infdis/173.6.1408.
34. Schroeder, N., Henry, T., de Chastellier, C., Zhao, W., Guilhon, A.A., Gorvel, J.P., and Méresse, S. (2010). The virulence protein SopD2 regulates membrane dynamics of Salmonella-containing vacuoles. PLoS Pathog. *6*, e1001002. https://doi.org/10.1371/journal.ppat.1001002.
35. Shea, J.E., Hensel, M., Gleeson, C., and Holden, D.W. (1996). Identification of a virulence locus encoding a second type III secretion system in Salmonella typhimurium. Proc. Natl. Acad. Sci. USA *93*, 2593–2597. https://doi.org/10.1073/pnas.93.6.2593.
36. Forest, C.G., Ferraro, E., Sabbagh, S.C., and Daigle, F. (2010). Intracellular survival of Salmonella enterica serovar Typhi in human macrophages is independent of Salmonella pathogenicity island (SPI)-2. Microbiology *156*, 3689–3698.
37. García Véscovi, E., Soncini, F.C., and Groisman, E.A. (1996). Mg2+ as an extracellular signal: environmental regulation of Salmonella virulence. Cell *84*, 165–174.
38. Bader, M.W., Sanowar, S., Daley, M.E., Schneider, A.R., Cho, U., Xu, W., Klevit, R.E., Le Moual, H., and Miller, S.I. (2005). Recognition of antimicrobial peptides by a bacterial sensor kinase. Cell *122*, 461–472.
39. Alpuche Aranda, C.M., Swanson, J.A., Loomis, W.P., and Miller, S.I. (1992). *Salmonella* Typhimurium activates virulence gene transcription within acidified macrophage phagosomes. Proc. Natl. Acad. Sci. USA *89*, 10079–10083. https://doi.org/10.1073/pnas.89.21.10079.
40. Prost, L.R., Daley, M.E., Le Sage, V., Bader, M.W., Le Moual, H., Klevit, R.E., and Miller, S.I. (2007). Activation of the bacterial sensor kinase PhoQ by acidic pH. Mol. Cell *26*, 165–174. https://doi.org/10.1016/j.molcel.2007.03.008.
41. Powers, T.R., Haeberle, A.L., Predeus, A.V., Hammarlöf, D.L., Cundiff, J.A., Saldaña-Ahuactzi, Z., Hokamp, K., Hinton, J.C.D., and Knodler, L.A. (2021). Intracellular niche-specific profiling reveals transcriptional adaptations required for the cytosolic lifestyle of Salmonella enterica. PLoS Pathog. *17*, e1009280. https://doi.org/10.1371/journal.ppat.1009280.
42. Röder, J., Felgner, P., and Hensel, M. (2021). Comprehensive single cell analyses of the nutritional environment of intracellular Salmonella enterica. Front. Cell. Infect. Microbiol. *11*, 624650. https://doi.org/10.3389/fcimb.2021.624650.
43. Kapetanovic, R., Bokil, N.J., Achard, M.E.S., Ong, C.-L.Y., Peters, K.M., Stocks, C.J., Phan, M.-D., Monteleone, M., Schroder, K., Irvine, K.M., et al. (2016). Salmonella employs multiple mechanisms to subvert the TLR-inducible zinc-mediated antimicrobial response of human macrophages. FASEB J. *30*, 1901–1912.
44. Dong, X.P., Cheng, X., Mills, E., Delling, M., Wang, F., Kurz, T., and Xu, H. (2008). The type IV mucolipidosis-associated protein TRPML1 is an endolysosomal iron release channel. Nature *455*, 992–996. https://doi.org/10.1038/nature07311.
45. Chen, C.C., Krogsaeter, E., Butz, E.S., Li, Y., Puertollano, R., Wahl-Schott, C., Biel, M., and Grimm, C. (2020). TRPML2 is an osmo/mechanosensitive cation channel in endolysosomal organelles. Sci. Adv. *6*, eabb5064. https://doi.org/10.1126/sciadv.abb5064.
46. Plesch, E., Chen, C.C., Butz, E., Scotto Rosato, A., Krogsaeter, E.K., Yinan, H., Bartel, K., Keller, M., Robaa, D., Teupser, D., et al. (2018). Selective agonist of TRPML2 reveals direct role in chemokine release from innate immune cells. Elife *7*, e39720. https://doi.org/10.7554/eLife.39720.

47. Chen, C.C., Cang, C., Fenske, S., Butz, E., Chao, Y.K., Biel, M., Ren, D., Wahl-Schott, C., and Grimm, C. (2017). Patch-clamp technique to characterize ion channels in enlarged individual endolysosomes. Nat. Protoc. *12*, 1639–1658. https://doi.org/10.1038/nprot.2017.036.

48. Dong, X.P., Shen, D., Wang, X., Dawson, T., Li, X., Zhang, Q., Cheng, X., Zhang, Y., Weisman, L.S., Delling, M., and Xu, H. (2010). PI(3,5)P(2) controls membrane trafficking by direct activation of mucolipin Ca(2+) release channels in the endolysosome. Nat. Commun. *1*, 38. https://doi.org/10.1038/ncomms1037.

49. Hernandez, L.D., Hueffer, K., Wenk, M.R., and Galán, J.E. (2004). Salmonella modulates vesicular traffic by altering phosphoinositide metabolism. Science *304*, 1805–1807. https://doi.org/10.1126/science.1098188.

50. Walpole, G.F.W., Pacheco, J., Chauhan, N., Clark, J., Anderson, K.E., Abbas, Y.M., Brabant-Kirwan, D., Montaño-Rendón, F., Liu, Z., Zhu, H., et al. (2022). Kinase-independent synthesis of 3-phosphorylated phosphoinositides by a phosphotransferase. Nat. Cell Biol. *24*, 708–722. https://doi.org/10.1038/s41556-022-00895-y.

51. Cirillo, D.M., Valdivia, R.H., Monack, D.M., and Falkow, S. (1998). Macrophage-dependent induction of the Salmonella pathogenicity island 2 type III secretion system and its role in intracellular survival. Mol. Microbiol. *30*, 175–188.

52. Dunstan, S.J., Hue, N.T., Han, B., Li, Z., Tram, T.T.B., Sim, K.S., Parry, C.M., Chinh, N.T., Vinh, H., Lan, N.P.H., et al. (2014). Variation at HLA-DRB1 is associated with resistance to enteric fever. Nat. Genet. *46*, 1333–1336. https://doi.org/10.1038/ng.3143.

53. Cunrath, O., and Bumann, D. (2019). Host resistance factor SLC11A1 restricts Salmonella growth through magnesium deprivation. Science *366*, 995–999.

54. Malo, D., Vogan, K., Vidal, S., Hu, J., Cellier, M., Schurr, E., Fuks, A., Bumstead, N., Morgan, K., and Gros, P. (1994). Haplotype mapping and sequence analysis of the mouse Nramp gene predict susceptibility to infection with intracellular parasites. Genomics *23*, 51–61. https://doi.org/10.1006/geno.1994.1458.

55. Yeom, J., Shao, Y., and Groisman, E.A. (2020). Small proteins regulate Salmonella survival inside macrophages by controlling degradation of a magnesium transporter. Proc. Natl. Acad. Sci. USA *117*, 20235–20243. https://doi.org/10.1073/pnas.2006116117.

56. Rinkenberger, N., and Schoggins, J.W. (2018). Mucolipin-2 cation channel increases trafficking efficiency of endocytosed viruses. mBio *9*, e02314-17.

57. Jaslow, S.L., Gibbs, K.D., Fricke, W.F., Wang, L., Pittman, K.J., Mammel, M.K., Thaden, J.T., Fowler, V.G., Hammer, G.E., Elfenbein, J.R., and Ko, D.C. (2018). *Salmonella* activation of STAT3 signaling by SarA effector promotes intracellular replication and production of IL-10. Cell Rep. *23*, 3525–3536.

58. Grimm, C., Jörs, S., Saldanha, S.A., Obukhov, A.G., Pan, B., Oshima, K., Cuajungco, M.P., Chase, P., Hodder, P., and Heller, S. (2010). Small molecule activators of TRPML3. Chem. Biol. *17*, 135–148. https://doi.org/10.1016/j.chembiol.2009.12.016.

59. Pujol, C., and Bliska, J.B. (2003). The ability to replicate in macrophages is conserved between *Yersinia pestis* and *Yersinia pseudotuberculosis*. Infect. Immun. *71*, 5892–5899. https://doi.org/10.1128/iai.71.10.5892-5899.2003.

60. Wang, R.F., and Kushner, S.R. (1991). Construction of versatile low-copy-number vectors for cloning, sequencing and gene expression in Escherichia coli. Gene *100*, 195–199.

61. Datsenko, K.A., and Wanner, B.L. (2000). One-step inactivation of chromosomal genes in Escherichia coli K-12 using PCR products. Proc. Natl. Acad. Sci. USA *97*, 6640–6645. https://doi.org/10.1073/pnas.120163297.

62. Cherepanov, P.P., and Wackernagel, W. (1995). Gene disruption in Escherichia coli: TcR and KmR cassettes with the option of Flp-catalyzed excision of the antibiotic-resistance determinant. Gene *158*, 9–14.

63. Chang, C.C., Chow, C.C., Tellier, L.C., Vattikuti, S., Purcell, S.M., and Lee, J.J. (2015). Second-generation PLINK: rising to the challenge of larger and richer datasets. Gigascience *4*, 7. https://doi.org/10.1186/s13742-015-0047-8.

64. Chen, S., Zhou, Y., Chen, Y., and Gu, J. (2018). fastp: an ultra-fast all-in-one FASTQ preprocessor. Bioinformatics *34*, i884–i890. https://doi.org/10.1093/bioinformatics/bty560.

65. Liao, Y., Smyth, G.K., and Shi, W. (2014). featureCounts: an efficient general purpose program for assigning sequence reads to genomic features. Bioinformatics *30*, 923–930. https://doi.org/10.1093/bioinformatics/btt656.

66. Love, M.I., Huber, W., and Anders, S. (2014). Moderated estimation of fold change and dispersion for RNA-seq data with DESeq2. Genome Biol. *15*, 550. https://doi.org/10.1186/s13059-014-0550-8.

67. Dobin, A., Davis, C.A., Schlesinger, F., Drenkow, J., Zaleski, C., Jha, S., Batut, P., Chaisson, M., and Gingeras, T.R. (2013). STAR: ultrafast universal RNA-seq aligner. Bioinformatics *29*, 15–21. https://doi.org/10.1093/bioinformatics/bts635.

68. Subramanian, A., Tamayo, P., Mootha, V.K., Mukherjee, S., Ebert, B.L., Gillette, M.A., Paulovich, A., Pomeroy, S.L., Golub, T.R., Lander, E.S., and Mesirov, J.P. (2005). Gene set enrichment analysis: a knowledge-based approach for interpreting genome-wide expression profiles. Proc. Natl. Acad. Sci. USA *102*, 15545–15550. https://doi.org/10.1073/pnas.0506580102.

69. Conant, D., Hsiau, T., Rossi, N., Oki, J., Maures, T., Waite, K., Yang, J., Joshi, S., Kelso, R., Holden, K., et al. (2022). Inference of CRISPR edits from sanger trace data. CRISPR J. *5*, 123–130. https://doi.org/10.1089/crispr.2021.0113.

70. Pruim, R.J., Welch, R.P., Sanna, S., Teslovich, T.M., Chines, P.S., Gliedt, T.P., Boehnke, M., Abecasis, G.R., and Willer, C.J. (2010). LocusZoom: regional visualization of genome-wide association scan results. Bioinformatics *26*, 2336–2337. https://doi.org/10.1093/bioinformatics/btq419.

71. 1000 Genomes Project Consortium; Auton, A., Brooks, L.D., Durbin, R.M., Garrison, E.P., Kang, H.M., Korbel, J.O., Marchini, J.L., McCarthy, S., McVean, G.A., and Abecasis, G.R. (2015). A global reference for human genetic variation. Nature *526*, 68–74. https://doi.org/10.1038/nature15393.

72. International HapMap Consortium (2005). A haplotype map of the human genome. Nature *437*, 1299–1320. https://doi.org/10.1038/nature04226.

73. Purcell, S., Neale, B., Todd-Brown, K., Thomas, L., Ferreira, M.A.R., Bender, D., Maller, J., Sklar, P., de Bakker, P.I.W., Daly, M.J., and Sham, P.C. (2007). PLINK: a tool set for whole-genome association and population-based linkage analyses. Am. J. Hum. Genet. *81*, 559–575. https://doi.org/10.1086/519795.

74. Wickham, H. (2016). ggplot2: Elegant Graphics for Data Analysis.

75. Arnold, J.B. (2021). Ggthemes: Extra Themes, Scales and Geoms for 'ggplot2'.

76. 1000 Genomes Project Consortium; Abecasis, G.R., Altshuler, D., Auton, A., Brooks, L.D., Durbin, R.M., Gibbs, R.A., Hurles, M.E., and McVean, G.A. (2010). A map of human genome variation from population-scale sequencing. Nature *467*, 1061–1073. https://doi.org/10.1038/nature09534.

77. Westermann, A.J., and Vogel, J. (2018). Host-pathogen transcriptomics by dual RNA-seq. In Bacterial Regulatory RNA: Methods and Protocols, V. Arluison and C. Valverde, eds. (Springer Sciences), pp. 59–75.

78. Kersey, P.J., Staines, D.M., Lawson, D., Kulesha, E., Derwent, P., Humphrey, J.C., Hughes, D.S.T., Keenan, S., Kerhornou, A., Koscielny, G., et al. (2012). Ensembl Genomes: an integrative resource for genome-scale data from non-vertebrate species. Nucleic Acids Res. *40*, D91–D97. https://doi.org/10.1093/nar/gkr895.

79. Mootha, V.K., Lindgren, C.M., Eriksson, K.F., Subramanian, A., Sihag, S., Lehar, J., Puigserver, P., Carlsson, E., Ridderstråle, M., Laurila, E., et al. (2003). PGC-1alpha-responsive genes involved in oxidative

CellPress
OPEN ACCESS

phosphorylation are coordinately downregulated in human diabetes. Nat. Genet. *34*, 267–273. https://doi.org/10.1038/ng1180.

80. Gasnier, B., Zhu, M.X., and Taylor & Francis. Ion and molecule transport in lysosomes. Methods in Signal Transduction. First edition. ed.CRC Press

81. Chen, C.C., Butz, E.S., Chao, Y.K., Grishchuk, Y., Becker, L., Heller, S., Slaugenhaupt, S.A., Biel, M., Wahl-Schott, C., and Grimm, C. (2017). Small molecules for early endosome-specific patch clamping. Cell Chem. Biol. *24*, 907–916.e4. https://doi.org/10.1016/j.chembiol.2017.05.025.

82. Harrell, F.E., Jr. (2021). Hmisc: Harrell Miscellaneous.

83. Wickham, H., François, R., Henry, L., and Müller, K. (2022). Dplyr: A Grammar of Data Manipulation.

STAR★METHODS

KEY RESOURCES TABLE

REAGENT or RESOURCE	SOURCE	IDENTIFIER
**Bacterial and virus strains**		
S. enterica Typhi Ty2 +p67GFP3.1	Dennis Ko[7]	DCK33
S. enterica Typhi Ty2 Δ*ssaT* + p67GFP3.1	This paper	DCK723
S. enterica Typhi Ty2 Δ*mgtA*Δ*mgtB* + p67GFP3.1	This paper	DCK1122
Salmonella enterica Typhi Ty2 with 956bp deletion in *phoPQ* +p67GFP3.1	Samuel Miller[33]	Ty800 or CS021
S. enterica Typhimurium 14028s +p67GFP3.1	Dennis Ko[5]	DCK22
S. enterica Typhimurium 14028s Δ*mgtA*Δ*mgtB* + p67GFP3.1	This paper	DCK1121
S. enterica Typhimurium 14028s Δ*mgtA*Δ*mgtB* + pWSK129	This paper	DCK1132
S. enterica Typhimurium 14028s +pWSK29	Dennis Ko[57]	DCK483
**Chemicals, peptides, and recombinant proteins**		
Accell siRNA delivery media	Horizon	B-005000
Accell non-targeting #1 (NT1) siRNA	Horizon	D-001910-01
Accell SmartPool *MCOLN2* siRNA	Horizon	E−021616-00
Accell SmartPool *MCOLN3* siRNA	Horizon	E−015371-00
Recombinant human IFN-γ	Peprotech	300–02
Recombinant human IFN-β	Peprotech	300-02BC
S. Typhimurium S-form LPS	Enzo	ALX-581-011
RNA*later* Solution	ThermoFisher	AM7020
Gentamicin Sulfate	VWR	45000–634
7-aminoactinomycin D (7AAD)	Enzo	ALX-380-283
$MgCl_2$ Hexahydrate BioReagent	Sigma	M2393
$ZnSO_4$ Heptahydrate BioReagent	Sigma	Z0251
TaqMan FAM-MGB *MCOLN2* probe	ThermoFisher	4331182 - Hs00401920
TaqMan FAM-MGB *MCOLN3* probe	ThermoFisher	4331182 - Hs00962657
Isopropyl ß-D-1-thiogalactopyranoside (IPTG)	ThermoFisher	15529–019
Hs TRPML2-YFP	Grimm et al.[58]	–
Transfection reagent TurboFect	Thermo Fisher	R0531
Vacuolin	Santa Cruz	sc-216045
PI(3,5)P2	AG Scientific	P-1123
ML2-SA1	Macro Keller and Franz Bracher	–
**Critical commercial assays**		
mirVana miRNA Isolation Kit	ThermoFisher	AM1560
TURBO DNase	ThermoFisher	AM2238
RNeasy MinElute cleanup Kit	Qiagen	74204
Standard Total RNA Prep with Ribo-Zero Plus	Ilumina	20037135
iTaq Universal SYBR Green Supermix	BioRad	1725124
RNeasy kit	Qiagen	74106
iScript cDNA Synthesis kit	BioRad	1708891
iTaq Universal Probes Supermix	BioRad	1725134
**Deposited data**		
Intracellular THP-1 RNA-seq	GEO	GSE222194
Cellular GWAS on Intracellular *S.* Typhi Replication	Duke Research Data Repository	https://doi.org/10.7924/r4x92bd76

(*Continued on next page*)

Continued

REAGENT or RESOURCE	SOURCE	IDENTIFIER
**Experimental models: Cell lines**		
Human THP-1 Monocytes: WT & *MCOLN2*$^{-/-}$ Pool	Synthego	RRID:CVCL_0006
Human: Lymphoblastoid Cell Lines (LCLs)	Coriell Institute	See Table S1 for all LCL individual identifiers
Human HEK 293	DSMZ	ACC 305
**Experimental models: Organisms/strains**		
Mus musculus: C57BL/6J *Mcoln2*$^{+/-}$	Rosa Puertollano	C57BL/6J
**Oligonucleotides**		
See Table S4 for list of primers.	This study	See Table S4
**Recombinant DNA**		
p67GFP3.1 (Amp^R, GFPmut3.1 under P_{tac})	Pujol & Bliska[59]	–
pWSK29 (Amp^R, very-low copy pSC101 ori)	Wang & Kushner[60]	Addgene #172972
pWSK129 (Kan^R, very-low copy pSC101 ori)	Wang & Kushner[60]	–
pKD4 (Amp^R, ts, FRT-Kan^R-FRT)	Datsenko & Wanner[61]	Addgene #45605
pKD46 (Amp^R, ts, λ red genes [*exo*, *bet*, *gam*] under P_{araB})	Datsenko & Wanner[61]	–
pCP20 (Amp^R, Cam^R, ts, *Flp*)	Cherepanov & Wackernagel[62]	–
**Software and algorithms**		
GraphPad Prism 9	GraphPad Software	www.graphpad.com
R 4.0.2	R Core Team	www.r-project.org
BioRender	BioRender	www.biorender.com
PLINK 1.9	Chang et al.[63]	www.cog-genomics.org/plink/
fastp: a FASTQ preprocessor	Chen et al.[64]	github.com/OpenGene/fastp
featureCounts tool	Liao et al.[65]	subread.sourceforge.net
DESeq2 Bioconductor	Love et al.[66]	bioconductor.org/packages/release/bioc/html/DESeq2.html
STAR RNA-seq alignment tool	Dobin et al.[67]	code.google.com/archive/p/rna-star/
GSEA 4.1	Subramanian et al.[68]	www.gsea-msigdb.org
ICE webtool 2.0	Conant et al.[69]	ice.synthego.com
LocusZoom webtool	Pruim et al.[70]	locuszoom.org

RESOURCE AVAILABILITY

Lead contact

Further information, as well as plasmids and bacterial strains generated for this study, are available by request from the lead contact, Dennis C. Ko (dennis.ko@duke.edu).

Materials availability

Plasmids and bacterial strains, as listed in the key resources table, are available upon request.

Data and code availability

Intracellular replication data for the 961 LCL samples can be found in Table S1, and GWAS summary statistics are available for download at the Duke Research Data Repository (Duke Research Data Repository: https://doi.org/10.7924/r4x92bd76). Intracellular *S.* Typhi RNA-seq data are available in GEO (GEO: GSE222194). The analyses of this data—differential gene expression and gene set enrichment analysis (GSEA)—are available in Table S2. In other replication experiments, the value of each biological replicate is shown as dots on top of bar graphs.

EXPERIMENTAL MODEL AND SUBJECT DETAILS

Human cells

Lymphoblastoid cell lines (LCLs; EBV-immortalized B cells) were from the Coriell Institute. *MCOLN2*$^{-/-}$ and matched wild-type THP-1 cell pools were generated by Synthego using guide 5′-TTTTGGTTTAAGTAACCAGC-3′ (PAM is TGG) to target the start of

MCOLN2 exon 3. THP-1 knock out pools were confirmed to maintain ≥85% frameshift indels by Sanger sequencing that was analyzed with the inference of CRISPR editing (ICE) webtool v2.0 (https://ice.synthego.com) from Synthego.[69] THP-1s and LCLs were maintained at 37°C in a 5% CO_2 atmosphere and were grown in RPMI 1640 media (Gibco #21870) supplemented with 10% heat-inactivated fetal bovine serum (HI-FBS, Gibco #10082), 2 mM L-glutamine (Gibco #25030081), & 100 U/mL Penicillin-Streptomycin (Gibco #15140122). Infection assays were carried out in the same media but without Pen-Strep and phenol red. Cells were verified as mycoplasma free by the Universal Mycoplasma Detection Kit (ATCC #30-1012K).

Mice

Mcoln2$^{+/-}$ C57BL/6J mice provided by Dr. Rosa Puertollano and maintained specific pathogen free by Duke DLAR breeding core in groups of 5 or less of the same sex post weaning. Sex of animals is denoted in figure legends. Mice were free fed standard diet (PicoLab Mouse Diet #5058) during infections. Infections were approved by Duke IACUC (protocol #A145-18-06).

Bacteria

S. enterica serovars Typhi strain Ty2, Typhimurium strain 14028s, and derived mutants were grown at 37°C and 250 rpm in high-salt Miller Luria-Bertani (LB) broth (VWR #90003). To quantify intracellular burden during gentamicin protection assays, Salmonellae were tagged with inducible GFP using p67GFP3.1,[59] which carries GFP under an IPTG-inducible promoter and is maintained with 100 mg/mL ampicillin. *Salmonella* gene deletion strains were generated by lambda-red recombineering[61] from Ty2 or 14028s using Kan^R cassettes generated from pKD4 with the primers in Table S4. Gene deletions were confirmed by PCR using indicated primers in Table S4.

METHOD DETAILS

Infection (gentamicin-protection) assays

Salmonella infection of LCL and THP-1 cells was done as previously described.[5] In brief, overnight stationary cultures in Miller LB were sub-cultured 1:33 and grown for 160 min at 37°C and 250 rpm to reach SPI-1 inducing late-log phase (an OD_{600} of 1.7–2.0 for *S.* Typhimurium and 0.8–1.1 for *S.* Typhi). 1×10^5 LCL or THP-1 cells were plated at 1×10^6 cells/mL in complete RPMI 1 h before infection in 96-well non-TC plates. LCLs were infected at multiplicity of infection (MOI) 30 and THP-1s at MOI 10. To kill the extra-cellular bacteria, gentamicin was added 1 h post infection (hpi) at 50 mg/mL and then diluted to 15 mg/mL at 2 hpi. In ion repletion experiments, 5μL of filter-sterilized $MgCl_2$ or $ZnSO_4$ in DI water, or water only control, was added to 200μL in 96-well plates immediately following gentamicin dilution at 2 hpi. To induce GFP in p67GFP3.1, 1.4 mM IPTG was added 75 min prior to the desired time point.

Invasion, pyroptosis, and initial burden were measured with a Guava EasyCyte Plus high-throughput flow cytometer (Millipore) at 3.5 hpi. Pyroptosis was quantified as the percent staining with 1 μg/mL 7AAD (7-aminoactinomycin D; Enzo Life Sciences). Invasion was quantified as the percent GFP$^+$ & 7AAD$^-$. Burden was quantified as median fluorescent intensity (MFI) of living (7AAD$^-$) and infected (GFP$^+$) cells. Intracellular replication (permissivity) was quantified by re-measuring burden at 24 hpi and taking the ratio of 24 hpi burden over initial 3.5 hpi burden.

Cellular GWAS

Hi-HoST screening of 961 LCLs from parent-offspring trios for *S.* Typhi intracellular replication occurred in two large sets. In one, *S.* Typhi intracellular replication was one of 79 host-pathogen phenotypes measured as part of the Hi-HoST Phenome Project (H2P2).[6] H2P2 measured replication in 527 LCLs from four population in the 1000 Genomes Project[71]: ESN (Esan in Nigeria), GWD (Gambians in Western Divisions in The Gambia), IBS (Iberian Population in Spain), and KHV (Kinh in Ho Chi Minh City, Vietnam). In this dataset, we determined that replication is a quantitative trait suitable for GWAS due to its inter-individual variation (mean of 1.7-fold with standard deviation of 0.3), high experimental repeatability (~75% variance is due to inter-individual variation in two-way ANOVA), and substantial heritability ($h^2 = 0.33$ with $p = 0.002$ in parent-offspring regression).[6] To these 527 LCLs, we added previously unpublished data on *S.* Typhi replication from 434 LCLs from four populations in the HapMap project: CEU (Utah residents with ancestry from northern and western Europe), YRI (Yoruba in Ibadan, Nigeria), CHB (Han Chinese in Beijing, China), and JPT (Japanese in Tokyo, Japan).[72] For all 961 LCLs, we used flow cytometry to quantify intracellular bacterial burden as the median fluorescent intensity (MFI) of GFP in infected host cells, which contain viable GFP-tagged *S.* Typhi (see above for details of this fluorescence-based gentamicin protection assay). From these MFI measurements, we calculated intracellular replication or permissivity as the ratio of 24 hpi to 3.5 hpi burden. Each LCL was measured on three sequential passages and the phenotype used for GWAS was calculated as the mean measurement of these three independent assays. Each batch of LCLs measured during Hi-HoST screening was *Z* score transformed to reduce inter-batch experimental variation: $Z = (x - \mu_{batch})/\sigma_{batch}$.

Genotypes were obtained from HapMap r28 and 1000 Genomes Project Phase 3 with imputation using 1000 Genomes Project Phase 3. Filters included minor allele frequency (MAF) < 0.05, SNP missingness of >0.2 and sample genotype missingness of >0.2, resulting in a total of 8,386,469 SNPs for subsequent analysis. Genome-wide association analysis was carried out using the QFAM-parents approach in PLINK v1.9[24,63,73] with adaptive permutations ranging from 1000 to a maximum of 10^9. The QFAM approach uses linear regression to test for association while separately permuting between and within family components to control

for family structure. The human genome reference assembly (GRCh37/hg19) was used for all analysis. QQ plots against neutral, χ^2, distribution were plotted using quantile-quantile function in R. Local Manhattan plots were generated using LocusZoom[70] webtool (http://locuszoom.org/). Linear regression of *Salmonella* replication by rs10873679 genotype was performed and plotted in R using ggplot2[74] & ggthemes[75] packages.

Human gene expression analyses

RNA-seq gene expression data of 448 LCLs from the 1000 Genomes Project[28] were obtained from the EBI website (https://www.ebi.ac.uk/gxa/experiments/E-GEUV-1/Downloads). The rs10873679 genotype data were downloaded from the 1000 genome project.[76] Effects of rs1087369 genotype on *MCOLN2* and *MCOLN3* gene expression in both datasets were tested by linear regression on combined data as well as individual populations and individual sexes. Protein abundance measured by isobaric tag-based quantitative mass-spectroscopy in 95 LCLs from HapMap project were obtained from Wu et al. However, only 33 of the individuals had quantifiable MCOLN2. The effect of rs10873679 on MCOLN2 protein abundance was tested by linear regression in R.

RNAi experiments and knockdown confirmation

LCLs or THP-1s (2.5×10^5 cells) were washed and re-suspended at 400,000 cells/mL in 500 μL of serum-free Accell siRNA delivery media (Horizon #B-005000) in TC-treated 24-well plates and treated for three days with 10 pg/μL of either Dharmacon Accell non-targeting #1 (NT1) (Horizon #D-001910-01) or an Accell SmartPool against human *MCOLN2* (Horizon #E−021616-00) or *MCOLN3* (Horizon #E−015371-00). Prior to infection, cells were washed and plated at 700,000 cells/mL in 100 μL complete RPMI media (without antibiotics) in 96-well non-TC plates. Infections were conducted as described above.

For each experiment, knockdown was confirmed by RT-qPCR. Briefly, RNA was extracted from one well not used for infection ($\sim 5 \times 10^5$ treated cells) for each siRNA condition using RNeasy kit (Qiagen #74106). Then cDNA was reverse transcribed from 500ng of RNA/condition using iScript kit (BioRad #1708891) and quantified by qPCR using iTaq Universal Probes Supermix (BioRad #1725134) and exon-spanning TaqMan FAM-MGB probes (ThermoFisher #4331182; *MCOLN2* is Hs00401920 & *MCOLN3* is Hs00962657) on a QuantStudio 3 thermocycler (ThermoFisher). All qPCR was run in technical triplicate. Mean comparative threshold cycle (C_T) value for each transcript was adjusted for input variation by subtracting the mean 18s (*RNA18S5*; ThermoFisher Hs03928990) housekeeping control C_T from the target gene's C_T to generate a ΔC_T. The $\Delta\Delta C_T$ for each knockdown was calculated by subtracting target gene ΔC_T in siNT1-treated control cells from target gene ΔC_T in siTarget-treated cells. Knockdown fold change was then calculated as $2^{-\Delta\Delta CT}$. Mean fold-change knockdown ± SEM was reported in figure legends.

Inducing and measuring *MCOLN2* expression

To measure *MCOLN2* induction, 5×10^5 THP-1s in 24-well non-TC treated plates were infected with *S*. Typhi Ty2 at MOI 10 following the above gentamicin-protection assay or stimulated 2 hpi with 500 U/mL (25 ng/mL) recombinant human IFN-γ (PeproTech #300-02) and 100 pg/mL well-vortexed *S*. Typhimurium S-form LPS (Enzo #ALX-581-011) or 50 U/mL (5 ng/mL) recombinant human IFN-β (PeproTech #300-02BC). At 24hpi, *MCOLN2* expression was measured by RT-qPCR following the same $\Delta\Delta C_T$ method used to measure knockdown.

Mouse infections

Litter and sex matched C57BL/6J mice bred from *Mcoln2*$^{+/-}$ parents by the Duke DLAR breeding core were infected when 10–18 weeks old with *S*. Typhimurium 14028s sub-cultured 1:33, grown for 160 min to late-log (OD_{600} 1.7–1.9), and then washed twice with sterile PBS. Bacteria were re-suspended in PBS at 10,000/mL based on OD_{600} and mice were infected via intraperitoneal injection with 100 μL of PBS containing 1,000 CFUs. For competitive infections, the initial 1:1 ratio used 500 CFUs of each Amp^R wild-type (+pWSK29; DCK483) and Kan^R mutant (Δ*mgtA*Δ*mgtB* + pWSK129; DCK1132) *S*. Typhimurum. All inoculums were verified by plating for CFUs. All mice were monitored twice daily for morbidity. Spleens were harvested 4 dpi, homogenized by bead beating with ZrO beads (GlenMills #7305-000031) with a Bead Ruptor 12 (Omni #19-050A), and a serial dilution was plated for CFUs on LB + Amp (100 μg/mL) or Kan (50 μg/mL). Competitive index was calculated as ratio of Kan/Amp CFUs.

Fluorescence-activated cell sorting (FACS)

For cell sorting RNA-seq samples, 20 million THP-1 monocytes of each *MCOLN2* genotype were plated into 24-well non-TC plates (500,000 cells per 0.5 mL RPMI per well) and infected with *S.* Typhi at MOI10. The remaining late-log *S.* Typhi inoculum was washed with PBS and fixed with 100 μL of RNA*later* Solution (ThermoFisher #AM7020) for 10 min at room temperature and then frozen for later RNA extraction. Following 2 h of IPTG induction, monocytes were spun down at 16 hpi and re-suspended at 10 million cells/mL in 2 mL of RPMI containing 15 μg/mL gentamicin and 1 μg/mL 7AAD. Two wells containing one million uninfected THP-1 monocytes of each genotype were washed with PBS and fixed in 1mL RNA*later* for use in the control.

Live monocytes were analyzed and sorted by the Duke Human Vaccine Institute (DHVI) flow cytometry shared resource using a FACSAria II (BD Biosciences) at 70 psi with at 70 μm nozzle. One million infected (GFP^+) and living ($7AAD^-$) cells were sorted into 1 mL of RNA*later* for immediate fixation and held at 4°C in a chilled collection tube rack. Doublets were excluded by FSC and SSC gating and a purity mask was applied to exclude droplets containing GFP^+ and GFP^- events.

S. Typhi infected THP-1 RNA extraction

After sorting, RNA was immediately isolated from collected cells using mirVana miRNA Isolation Kit's total RNA protocol (ThermoFisher #AM1560). Prior to extraction, samples in RNA*later* were diluted 3x with PBS, spun down at 5,000 xg, and aspirated to remove RNA*later*. After resuspending cells in the kit's L/B buffer, samples were vortexed for 60 s to ensure lysis of *Salmonella*. Following total RNA extraction, gDNA was removed with 4 U TURBO DNase (ThermoFisher #AM2238) per μg of RNA and then purified with RNeasy MinElute cleanup kit (Qiagen #74204). In the gDNA-free RNA, the relative human to bacterial mRNA ratio was determined by RT-qPCR measurement of human *ACTB* and *S*. Typhi *rpoD.* In brief, 150ng of RNA was reverse transcribed with iScript cDNA synthesis kit (Bio-Rad #1708891) and 1:5 dilution of this cDNA was analyzed using iTaq Universal SYBR Green Supermix (Bio-Rad #1725124) and primers listed in Table S4 on a QuantStudio 3 System (ThermoFisher). This ratio was used to combine uninfected THP-1 mRNA and late-log *S*. Typhi inoculum mRNA for the control samples following Westermann & Vogel's approach.[77]

THP-1 and intracellular *S*. Typhi Dual RNA-seq

RNA was isolated from three independent experiments and submitted to the Duke Sequencing and Genomic Technologies (SGT) Shared Resource for cDNA library preparation with Illumina Standard Total RNA Prep with Ribo-Zero Plus (Illumina #20037135). These rRNA-depleted libraries were sequenced on Ilumina NovaSeq 6000 S prime flow cell with 100 bp paired-end reads.

RNA-seq data were processed using the fastp toolkit[64] to trim low-quality bases and Illumina sequencing adapters from the 3′ end of the reads. Only reads that were ≥20nt after trimming were kept for further analysis. Reads were mapped to a custom genome reference combining the GRCh38v93 version of the human genome and transcriptome[78] with *S. enterica* serovar Typhi strain Ty2 ASM754v1 genome and transcriptome using the STAR RNA-seq alignment tool.[67] Reads were kept for subsequent analysis if they mapped to a single genomic location. Gene counts were compiled using the featureCounts tool.[65] Only genes that had at least 10 reads in any given library were used in subsequent analysis. Normalization and differential expression within each species was carried out using the DESeq25 Bioconductor[66] package with the R statistical programming environment. The FDR was calculated to control for multiple hypothesis testing.

S. Typhi gene set enrichment analysis (GSEA)

Intracellular *S*. Typhi RNA-seq results were converted into a ranked gene list by multiplying the $\log_2(p)$ by the sign of the $\log_2$ fold-change (expression inside KO/WT THP-1). Fifteen *S*. Typhi gene sets related to divalent cation transport or virulence were generated, as shown in Table S1, and analyzed using GSEA v4.1.[68,79]

Endolysosomal patch-clamp experiments

The protocol of whole-endolysosome recordings have been described previously in detail.[47,80] HEK-293 cells were plated onto poly-L-lysine (Sigma)-coated glass coverslips. Human TRPML2 WT was transiently transfected into HEK-293 cells using TurboFect Transfection Reagent (Thermo Fisher Scientific) for 16–24 h. Cells were treated with vacuolin-1, a lipid-soluble polycyclic triazine that selectively enlarges endolysosomes homotypically (HEK293 cells, 1 μM overnight) up to 2–5 μm (capacitance = 0.39 ± 0.01 pF, n = 12 vacuoles). Vacuolin-1 were washed out before patch-clamp experimentation. Electrophysiological data were recorded using EPC-10 patch-clamp amplifier (HEKA, Lambrech, Germany), Axonpatch 200B (Molecular Devices), PatchMaster acquisition software (HEKA) and pClamp v10 software (Molecular Devices). Ramp protocol (−100 mV to +100 mV in 500 ms, holding potential = 0 mV). The current amplitudes at −100 mV were extracted from individual ramp recordings. Data were digitized at 40 kHz and filtered at 2.8 kHz. The compensation of capacitive transients and liquid junction potential were corrected as described.[81] For the application of 10 μM diC8-PI(3,5)P_2 (AG Scientific) or 30 μM ML2-SA1 in the bath solutions, the perfusion system and direct bath application were performed. ML2-SA1 were kindly provided by Macro Keller and Franz Bracher.[46] The cytoplasmic solution (bath) contained 150 mM NMDG and 10 mM HEPES (pH 7.2). Luminal solution (pipette) contained 105 $MgCl_2$, 5 mM HEPES, and 5 mM MES (2-(N-Morpholino)-ethane sulfonic acid) (pH 7.2). All statistical analysis was done using Origin9 software.

QUANTIFICATION AND STATISTICAL ANALYSIS

Descriptive statistics were performed with GraphPad Prism v9 (GraphPad Software, US) or R v4.0.2 (R Core Team) using Hmisc[82] & dplyr[83] packages. All replication ratios were $\log_2$-transformed or z-scored by batch before analysis. The size of each study or number of replicates, along with the statistical tests performed can be found in figure legends. Unless otherwise indicated, all datasets passed normality tests indicating no significant deviation from a Gaussian distribution. *In vitro* inter-experimental variability was removed prior to data visualization or statistical analysis by making experimental means equal to the grand mean by multiplying all values within each experiment by a normalization constant. These constants were calculated by dividing the mean of all experiments by mean of each specific experiment. Bar graphs represented the mean ± SEM (standard error of mean), unless otherwise noted. If an outlier was removed, it is noted in the figure legend along with the original value and the test used to exclude it.

Cell Genomics, Volume *3*

Supplemental information

Human variation impacting *MCOLN2* restricts *Salmonella* Typhi replication by magnesium deprivation

Kyle D. Gibbs, Liuyang Wang, Zhuo Yang, Caroline E. Anderson, Jeffrey S. Bourgeois, Yanlu Cao, Margaret R. Gaggioli, Martin Biel, Rosa Puertollano, Cheng-Chang Chen, and Dennis C. Ko

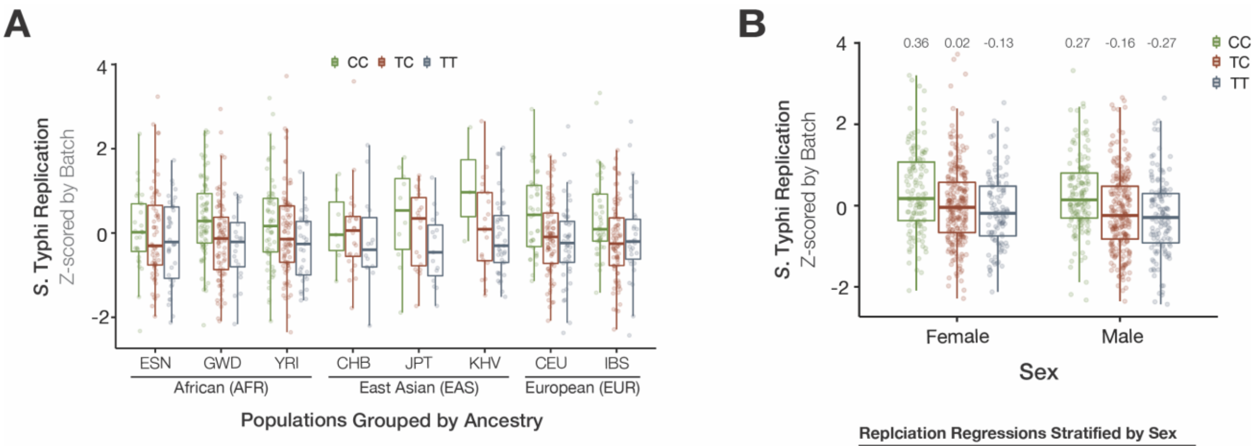

Populations in Hi-HoST Cellular GWAS

Ancestry	Pop	Description	N	N
	ESN	Esan in Nigeria	129	
AFR	GWD	Gambian Mandinka in Western Division, The Gambia	179	486
	YRI	Yoruba in Ibadan, Nigeria	178	
	CHB	Han Chinese in Beijing, China	45	
EAS	JPT	Japanese in Tokyo, Japan	45	152
	KHV	Kihn Vietnamese in Ho Chi Minh City, Vietnam	62	
EUR	CEU	Utah residents with N & W European ancestry	166	323
	IBS	Iberians in Spain	157	

Replciation Regressions Stratified by Sex

	Female	Male	All
N	460	501	961
Slope	-0.25	-0.28	-0.26
SE of Slope	±0.06	±0.06	±0.04
p value	1.3×10^{-4}	3.2×10^{-6}	1.7×10^{9}
R^2	0.029	0.041	0.036

Figure S1. The rs10873679 C-allele associates with more *S.* Typhi replication across all sampled populations and in both sexes, related to Figure 1. (**A**) Increased z-scored *S.* Typhi replication associated with the C-allele in all populations. Individual population regressions slopes range from -0.12 in ESN to -0.40 in GWD. Table below lists number of LCLs from each population used in cellular GWAS. (**B**) The rs10873679 C-allele associated with more z-scored *S.* Typhi replication in both sexes. Linear regressions are listed in the table. A two-way ANOVA with genotype and sex finds no significant interaction between sex & *MCOLN2* genotype ($p = 0.8$) despite both individual factors being a significant source of variation (genotype $p=1.9 \times 10^{-9}$ & sex $p=0.02$).

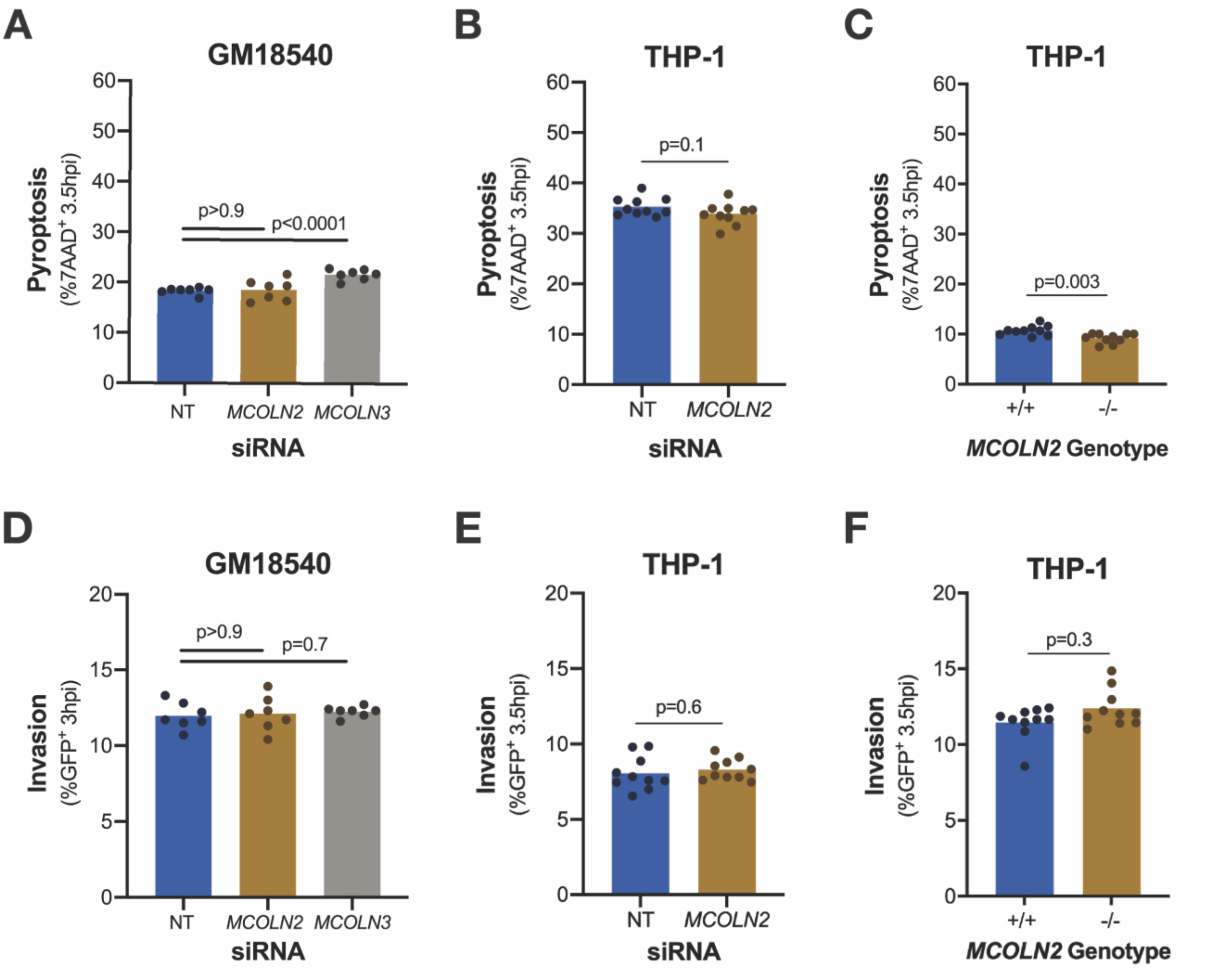

Figure S2. Neither *MCOLN2* knockdown nor knockout affect pyroptosis or invasion during *S.* Typhi infection in Figure 2E-G. (**A–C**) Pyroptosis is the percent of cells dead (7AAD^{+}) at 3.5 hpi. (**D–E**) Invasion is the percent of living (7AAD^{-}) and infected (GFP^{+}) cells at 3.5 hpi. LCL (GM18540) infection in A & D are nine replicates from three experiments from Figure 2E. THP-1 infection in B, C, E, & F are ten replicates from two experiments presented in Figure 2F & G. In A & F, the p values are from Mann-Whitney U tests. In B-E, p values are from unpaired *t* tests. While wild-type and knockout cells significantly differ in C, the effect size is only 0.5% respectively.

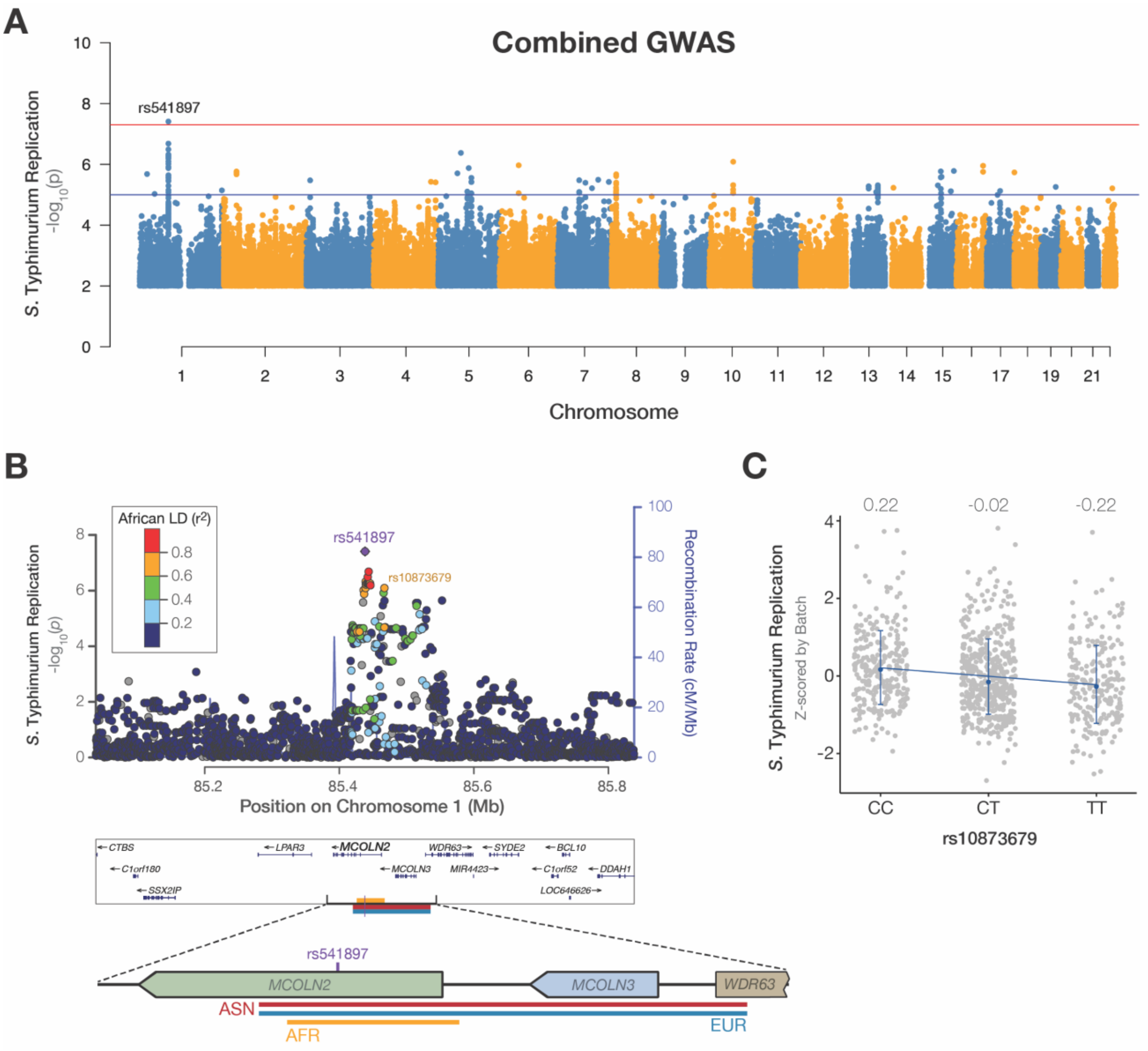

Figure S3. Increased *S.* Typhimurium replication also associates with the rs10873679 C- allele, related to Figure 2H. (**A**) The rs541897 locus association with z-scored *S.* Typhimurium replication ($p = 3.9x10^{-8}$) passes genome-wide significance threshold ($5x10^{-8}$; red line). **(B)** A local Manhattan plot of the *S.* Typhimurium replication-associated locus on chromosome 1 (hg19 build) shows that the *S.* Typhimurium lead SNP, rs541897, is in the same locus as the *S.* Typhi lead SNP, rs10873679. The *S.* Typhi and *S.* Typhimurium lead SNPs are linked (LD $r^2 = 0.71$ in African ancestry populations). Dots for each SNP are color coded by African (AFR) linkage disequilibrium (LD; r^2) from 1000 genomes Nov 2014 release. A 164 kb zoom in on the *MCOLN2/3* region indicates regions well-linked ($r^2>0.6$) with rs541897 by continental ancestry: African with a 41 kb orange bar, European (EUR) with a 116 kb blue bar, & Asian (ASN) with a 116 kb red bar. **(C)** The rs10873679 C-allele associates with more *S.* Typhimurium replication. The slope of a linear regression predicting replication from genotype is significantly different from zero ($\beta = -0.22 \pm 0.04$; $p = 3.5x10^{-6}$). Mean replication of each genotype is listed above their respective dot plot. Bars are mean ±SD.

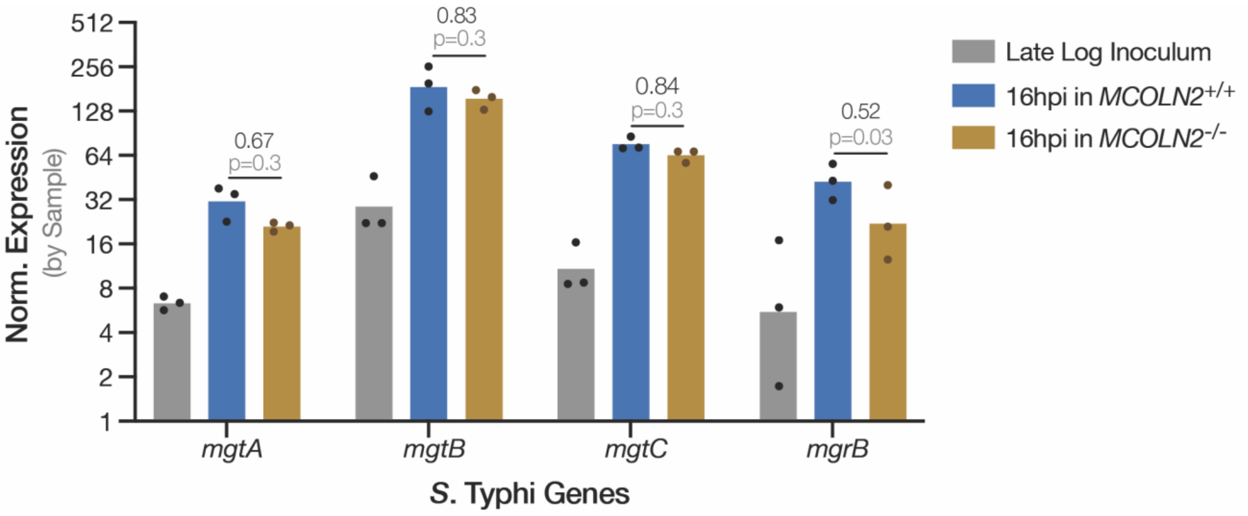

Figure S4. The increased expression of Mg^{2+}-regulated *S.* Typhi genes inside THP-1 monocytes is consistently decreased in *MCOLN2* knockouts, same data underlying log_2[Fold Change] in Figure 3D. Three replicates are from three experiments. Bars are geometric mean. Inoculum RNA was mixed with RNA from uninfected THP-1 cells of each genotype, so each late log replicate is the average of two technical (sequencing) replicates. Ratios (black text) are geometric mean from expression of bacteria replicating inside *MCOLN2* knockout over bacteria replicating in wild-type THP-1 cells. P values (grey) are from paired t tests corrected for multiple testing by Holm-Šídák method.

Table S3. *Salmonella enterica* ser. Typhi strain Ty2 gene sets used in GSEA. Related to Figures 3C and 3D.

Gene Sets	N	Genes																							
**Housekeeping**	**9**	*rpoD*	*aroC*	*dnaN*	*hemD*	*hisD*	*sucA*	*thrA*	*dnaK*	*rpoA*															
**SPI-1**	**24**	*sicP*	*sptP*	*iacP*	*prgH*	*invA*	*sigE*	*invF*	*spaM*	*prgI*	*invF*	*sipA*	*sipB*	*sipC*	*sipD*	*spaS*	*spaR*	*spaP*	*spaO*	*hilA*	*hilD*	*spaK*	*spaJ*	*spaI*	*spaH*
**SPI-2**	**23**	*spiC*	*spiA*	*ssaD*	*ssaE*	*sseA*	*sseB*	*sscA*	*sseC*	*sseD*	*sseE*	*sscB*	*sseF*	*sseG*	*ssaG*	*ssaI*	*ssaJ*	*ssaK*	*ssaL*	*ssaR*	*ssaR*	*ssaS*	*ssaT*	*ssaU*	
**PhoP Regulon**	**19**	*pmrD*	*mgtA*	*phoN*	*phoP*	*phoQ*	*mgtB*	*virK*	*ybjX*	*mgrB*	*pagP*	*pagD*	*iraP*	*pagC*	*mgtC*	*pipD*	*pagK*	*ugtL*	*pgtE*	*spiC*					
**Magnesium Influx**	**4**	*mgtA*	*mgtB*	*mgtS*	*mgrB*																				
**PmrA Regulon**	**11**	*pmrC*	*pmrA*	*pmrB*	*naxD*	*arnB*	*arnC*	*arnA*	*arnD*	*arnT*	*arnE*	*arnF*													
**Zinc Efflux**	**4**	*fieF*	*zntA*	*zntB*	*zitB*																				
**Zinc Influx**	**4**	*zupT*	*znuA*	*znuB*	*znuC*																				
**Ferrous Iron Efflux**	**2**	*fieF*	*iceT*																						
**Ferrous Iron Influx**	**3**	*feoA*	*feoB*	*feoC*																					
**Manganese Influx**	**7**	*mntH*	*sitA*	*sitB*	*sitC*	*sitD*	*mntR*	*mntS*																	
**Copper Response**	**8**	*copA*	*cueR*	*cueO*	*cueP*	*scsA*	*scsB*	*scsC*	*scsD*																
**MFS Efflux**	**5**	*mdtH*	*mdtM*	*mdtG*	*emrD*	*mdfA*																			
**TolC Efflux**	**5**	*macB*	*tolC*	*acrA*	*acrE*	*marA*																			
**RpoE Regulon**	**5**	*rpoE*	*rseA*	*rseB*	*rseC*																				

Table S4. Oligonucleotides grouped by function in study. Related to STAR Methods.

Lambda-Red Recombination

Target Gene	Gene ID	Primer Designations	Fwd for Kan^R Cassette Generation	Rvr for Kan^R Cassette Generation
mgtA	t4491	DK972 & DK973	TATAATCCGCGGCGCAAATTATTTACTTACCGGAGGCGAC gtgtaggctggagctgcttc	TCGGGGATTAAGCACGCTGGCGAATCCCCGACGAAAGTGT catatgaatatcctccttag
mgtB	t3755	DK974 & DK975	ATATGCAGGAAACACTACACCTTAATTTTGGGGATTCATC gtgtaggctggagctgcttc	TATCGGGTGAGCGATTCATCTGGGCGATCCTCAAACATTA catatgaatatcctccttag
ssaT	t1289	DK3 & DK433	tacccggcagataatgttacgaattggagagcatggttga gtgtaggctggagctgcttc	TCACGTAATTTCTTTTCTGTAGGCTGTTCTGTTTTCTCGC catatgaatatcctccttag

Deletion Confirmation

Target Gene(s)	Gene ID	Primer Designations	Fwd	Rvr
phoPQ	t1689 & t1690	DK1008 & DK1009	CATGACGCCGGCAAATTATATC	GAAAGTCGGGCCAGTTAAGA
ssaT	t1289	DK434 & DK435	CGGTAGTTGGTGTCATCGTAAG	AGCGCAATCAGCTGAAATAATG
mgtA	t4491	DK980 & DK981	CGTGACGCTGATGGTGATAAA	ACATCTCCTCCTCTCGTTCTG
mgtB	t3755	DK982 & DK983	CAGGCGTATAAGGAGGGAATG	ACACCAACAGGCTAATCAGTAA
MCOLN2	ENSG00000153898	DK859 & DK860	CTGGGGTAATTTTCCAAAAGCAGT	AGGTTATTCTTCTGTGGGACTTGT

SYBR RT-qPCR

Target Gene(s)	Gene ID	Primer Designations	Fwd	Rvr
ACTB	ENSG00000075624	DK880 & DK881	CCTGTACGCCAACACAGTGC	ATACTCCTGCTTGCTGATCC
rpoD	t3131	DK628 & DK629	GAAATGGGCACTGTTGAACTG	CAGATAGGTAATGGCTTCCGG
